# Weight change and long-term cardiovascular outcomes in overweight and obese patients after acute coronary syndromes

**DOI:** 10.1093/ehjopen/oeag105

**Published:** 2026-06-09

**Authors:** Louise Artels, Cédric Follonier, Stéphanie Baggio, David Carballo, David Nanchen, Kostantinos Koskinas, Christian M Matter, Lorenz Räber, Olivier Muller, Nicolas Rodondi, François Mach, Baris Gencer

**Affiliations:** Department of Cardiology, Lausanne University Hospitals, Rue du Bugnon 46, Lausanne 1011, Switzerland; Department of General Internal Medicine and Cardiology, Geneva University Hospitals, Rue Gabrielle-Perret-Gentil 4, Geneva 1205, Switzerland; Institute of Primary Health Care (BIHAM), University of Bern, Mittelstrasse 43, Bern 3012, Switzerland; Institute of Psychology, University of Lausanne, Geopolis, Chavannes-près-Renens 1015, Switzerland; Department of Cardiology, Geneva University Hospitals, Rue Gabrielle-Perret-Gentil 4, Geneva 1205, Switzerland; Department of Ambulatory Care and Community Medicine, Lausanne University, Route de la Corniche 21, Lausanne 1010, Switzerland; Department of Cardiology, Inselspital, Bern University Hospital, Freiburgstrasse 18, Bern 3010, Switzerland; Department of Cardiology, University Heart Center, University of Zurich, Rämistrasse 100, Zurich 8091, Switzerland; Department of Cardiology, Inselspital, Bern University Hospital, Freiburgstrasse 18, Bern 3010, Switzerland; Department of Cardiology, Lausanne University Hospitals, Rue du Bugnon 46, Lausanne 1011, Switzerland; Institute of Primary Health Care (BIHAM), University of Bern, Mittelstrasse 43, Bern 3012, Switzerland; Department of General Internal Medicine, Inselspital, Bern University Hospital, University of Bern, Freiburgstrasse 18, Bern 3010, Switzerland; Department of Cardiology, Geneva University Hospitals, Rue Gabrielle-Perret-Gentil 4, Geneva 1205, Switzerland; Department of Cardiology, Lausanne University Hospitals, Rue du Bugnon 46, Lausanne 1011, Switzerland

**Keywords:** Myocardial infarction, Secondary prevention, Body weight, Obesity, Lifestyle

## Abstract

**Aims:**

Overweight and obesity are common among patients with acute coronary syndromes (ACS), yet the long-term cardiovascular consequences of post-ACS weight change remain uncertain. We investigated the association between 1-year weight change after ACS and 5-year clinical outcomes in overweight and obese patients.

**Methods and results:**

We analysed 1473 overweight and obese ACS participants from the Swiss ELIPS cohort with available weight measurements. Hazard ratios (HRs) were estimated per 1% and 5% weight change for the 5-year risk of major adverse cardiovascular events (MACE), defined as cardiovascular death, non-fatal myocardial infarction, coronary revascularization, stroke, or transient ischaemic attack. Models were adjusted for age, sex, body mass index (BMI), smoking, total cholesterol, hypertension, diabetes, and estimated glomerular filtration rate. Predefined subgroups included BMI ≥ 27 kg/m^2^ vs. 25–27 kg/m^2^. Among the participants (15.1% women, mean age 61.5 years), the median BMI was 28.1 kg/m^2^ (67% BMI ≥ 27 kg/m^2^). Median 1-year weight change was 0%; 47.5% lost weight and 18.7% achieved >5% weight loss. Patient characteristics associated with weight loss were older age, higher BMI, non-smoking status, and participation in cardiac rehabilitation. During follow-up, 278 MACE events occurred. Weight loss was associated with a lower risk of MACE [multivariate-adjusted HR per 5%: 0.90, 95% confidence interval (CI): 0.82–1.00, *P* = 0.04]. The association was stronger in patients with BMI ≥ 27 kg/m^2^ (adj HR per 5% 0.87, 95% CI: 0.77–0.97) compared to those with BMI 25–27 kg/m^2^ (adj HR per 5% 1.01, 95% CI: 0.85–1.20, interaction *P* = 0.16).

**Conclusion:**

Overweight and obese ACS patients who lose weight may have a lower risk of cardiovascular events.

## Graphical Abstract

**Graphical Abstract:**
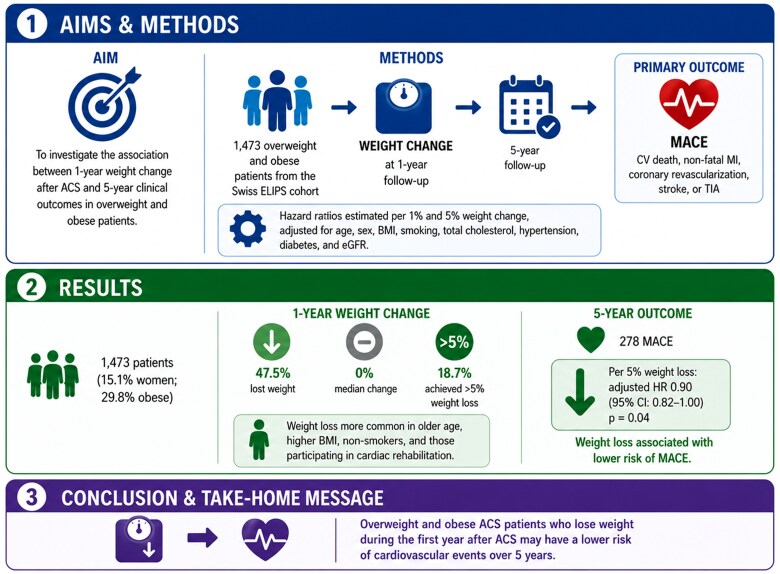

## Introduction

Overweight and obesity are common among patients following acute coronary syndromes (ACS), with prevalence estimates ranging from 60 to 80% in representative patient cohorts.^1–3^ Obesity is a well-established risk factor for cardiovascular disease (CVD), contributing to its development and progression through a complex interplay of metabolic, endocrine, inflammatory, structural, and haemodynamic alterations.^4–7^ In obesity, particularly the increased visceral fat promotes insulin resistance, dyslipidaemia, hypertension, and systemic inflammation.^7^ The 2021 European Society of Cardiology (ESC) guidelines on CVD prevention and the 2023 guidelines on the management of ACS recommend that overweight and obese people aim for a weight reduction of 5–10% to improve their CVD risk profile. However, available data suggest that weight reduction in real-life settings after ACS is suboptimal, with only 19.5% of obese patients losing ≥5% of their initial weight.^3,8^

In line with this evidence, the 2023 ESC guidelines recommend the use of glucagon-like peptide-1 receptor agonists (GLP-1 RAs) with proven cardiovascular benefit for secondary prevention in patients with type 2 diabetes and established atherosclerotic CVD, irrespective of glycaemic control or concurrent glucose-lowering therapy (Class I, Level A). Moreover, GLP-1 RAs should be considered to support weight loss in individuals with overweight or obesity (Class IIa, Level B). Recent recommendations are based on large-scale trials that have demonstrated cardiovascular benefits of GLP-1 RA, in individuals with overweight or obesity.^9,10^ In the SELECT trial, semaglutide in individuals with body mass index (BMI) ≥ 27 kg/m^2^ and established CVD without diabetes resulted in a placebo-adjusted mean weight reduction of 8.5% and a 20% relative reduction in major adverse cardiovascular events (MACE) over a mean follow-up of 39.8 months.^11^ These findings underscore the benefit of pharmacologically based weight-loss strategies on clinical outcomes. They also highlight the drug-based therapeutic opportunities for patients unable to achieve sufficient weight loss through lifestyle interventions alone. However, populations in cardiovascular drug trials are generally not representative of patients managed in real life.^12^

Currently, few studies have evaluated weight changes in overweight and obese patients after ACS outside of clinical trials, as well as their association with clinical outcomes. The relationship between body weight and outcomes after ACS remains complex. Observational studies and meta-analyses have described the so-called ‘obesity paradox’, whereby patients with overweight or moderate obesity appear to have lower mortality than those with normal BMI, while individuals with low BMI experience the highest risk.^13,14^ However, most of these studies relied on BMI measured at a single time point and did not evaluate longitudinal changes in body weight after the acute event. Consequently, whether post-ACS weight loss among overweight and obese patients is associated with improved or worsened cardiovascular outcomes remains insufficiently understood. In this context, our study specifically focused on longitudinal weight change among overweight and obese patients after ACS, rather than cross-sectional BMI categories or comparisons with participants of normal weight at baseline.

ELIPS is one of the few cohorts that captured weight changes over the year following ACS, while also measuring MACE and controlling cardiovascular risk factors over a 5-year follow-up period.^8,15^ The primary objective of this analysis was to evaluate the association between weight change during the first year after hospital discharge for ACS in overweight and obese patients and the risk of MACE and mortality at 5 years. The secondary objectives were to assess the association between weight changes and cardiovascular risk factor control and to identify clinical predictors of weight change.

## Methods

### Study design and setting

This study was a secondary analysis of the ELIPS cohort (NCT01075867), a prospective, multicentre observational study following patients after ACS that aimed to assess the quality of care and adherence to recommended preventive treatments at four academic Swiss medical centres (Bern, Geneva, Lausanne, and Zurich) embedded in the Special Program University Medicine-Acute Coronary Syndromes (SPUM-ACS) consortium (NCT01000701).

### Participants and procedures

Details on patient inclusion and follow-up have been described previously.^15–17^ In brief, men and women aged ≥18 years presenting with a primary diagnosis of ST-segment elevation myocardial infarction (STEMI), non-ST-segment elevation myocardial infarction (NSTEMI), or unstable angina (UA) were eligible for inclusion. Patients were excluded if they had severe physical disability, cognitive impairment, inability to provide informed consent, or a life expectancy < 1 year due to non-cardiac causes. The study protocol was approved by the local ethics committees (NCT01075867), and all participants provided written informed consent. For the present analysis, we included participants enrolled from 2009 to 2017 who completed both the 1-year and 5-year follow-up visits and who had baseline BMI ≥ 25 kg/m^2^ (definition of overweight).

In addition to weight measurement, study visits included, among other assessments, blood pressure (BP) measurement, laboratory analyses for LDL cholesterol (LDL-C), glycated haemoglobin (HbA1c), and fasting plasma glucose, and documentation of medication use. The use of GLP-1 RA was not specifically recorded, but their use was uncommon during the study period. Measurements were performed according to harmonized protocols across centres.^18,19^

### Exposure

The weight recorded at baseline was based on the measurement performed by healthcare providers following local standard practice during hospitalization. Weight was reported at 0.2 kg precision and measured without shoes. Weight change (%) was calculated as the percentage difference between follow-up and baseline weight: (follow-up weight − baseline weight)/baseline weight × 100. Participants with missing weight data were excluded from the primary analyses. Multiple imputation analyses including missing weight values were performed as sensitivity analyses. Weight loss was defined as a weight change per cent < 0% and weight gain or stable weight as a weight change per cent ≥ 0%.

### Outcomes

The primary outcome was a 4-point MACE defined as a composite of cardiovascular death, non-fatal myocardial infarction, coronary revascularization, stroke, or transient ischaemic attack.^20^ Secondary outcomes were a 3-point MACE defined as a composite of cardiovascular death, non-fatal myocardial infarction, stroke, or transient ischaemic attack,^11^ individual components of the composite and all-cause death. The MACE classification at the 5-year follow-up was adjudicated by a panel of independent clinicians using prespecified definitions and review of medical records.

### Statistical analyses

Descriptive statistics were reported according to weight change. Continuous variables are presented as means ± standard deviations (SDs), except for BMI and triglycerides, which are presented as medians with interquartile ranges (IQRs) due to skewed distributions. Group comparisons were performed using one-way ANOVA or Kruskal–Wallis tests, as appropriate. Categorical variables are summarized as counts and percentages, with group comparisons assessed using χ² tests. As this was a secondary analysis of an existing cohort, no sample size calculation was performed.

Weight trajectories were evaluated among participants with available body weight measurements at baseline, 1 year, and 5 years (*n* = 916). Descriptive analyses included mean weight change (%) ± SD over time according to the 1-year weight change group and were visualized graphically.

To identify predictors of weight loss, a linear regression model was fitted with percentage weight change as a continuous dependent variable. The model included the following independent variables based on clinical plausibility: sex,^21,22^ age (per 10-year),^23,24^ baseline BMI (per 5 kg/m^2^),^25^ education,^20^ current smoking status,^26^ alcohol consumption,^27^ baseline cholesterol (per 1 mmol/L),^28^ baseline eGFR (per 1 mL/min/1.73 m^2^),^29^ baseline diabetes,^30^ baseline hypertension,^31^ ACS type,^32^ and participation in cardiac rehabilitation.^33^ Model results were reported as b coefficients with 95% confidence intervals (CIs) and *P*-values.

We assessed the association between percentage weight change from baseline to 1 year and clinical cardiovascular outcomes between the 1- and 5-year follow-up using Cox regression. Participants were followed until the time of the first event, death, loss to follow-up, withdrawal, or at the final visit (maximum 6 years after discharge). Weight change was treated as a continuous predictor, with hazard ratios (HRs) calculated per 1% and 5% decrease. Both unadjusted and adjusted models were fitted. Adjusted models included age, sex, baseline BMI, smoking status, total cholesterol, hypertension, diabetes, and estimated glomerular filtration rate (eGFR).^34^ Results are presented as HRs with 95% CIs and *P*-values. In a sensitivity analysis, the models were also adjusted for participation in cardiac rehabilitation.

Predefined subgroup analyses comprised sex, age (<65 vs. ≥65), baseline BMI (25–27, ≥27 kg/m^2^ according to inclusion criteria for GLP-1 RA), smoking status (current vs. non-smoker), ACS type (STEMI vs. NSTEMI/UA), and cardiac rehabilitation programme participation after discharge. Adjusted Cox proportional hazards models were fitted within each subgroup, using the same covariates. To test for heterogeneity in associations, interaction terms between continuous weight change and subgroup variables were added to full models, and interaction *P*-values were derived from likelihood ratio tests.

To assess potential non-linear associations between percentage weight change and subsequent outcomes, we fitted Cox proportional hazards models adjusted for the same set of covariates. Weight change was modelled as a continuous variable using restricted cubic splines, with the degrees of freedom set to 3 after comparing alternative specifications for model fit and interpretability. We evaluated (i) the overall association of the spline term using a likelihood ratio test and (ii) non-linearity by comparing the spline model with an otherwise identical model including weight change as a linear term. Adjusted HRs with 95% CIs were estimated across the observed range of weight change, while holding all other covariates constant at reference values. The resulting curves were presented together with histograms illustrating the distribution of weight change in the study population. For comparison, equivalent multivariable Poisson regressions were also fitted to verify the consistency of results across modelling frameworks. Follow-up time was included as an offset, and incidence rates were expressed per 100 patient-years.

To assess the association between weight loss and achievement of 1-year cardiometabolic targets, we conducted logistic regression using percentage weight change as the exposure. For each outcome, we fitted unadjusted models, models adjusted for covariates, and models further adjusted for the baseline value of the outcome. Odds ratios (ORs) with 95% CIs were estimated per 1% and 5% weight loss. The preventive targets were defined based on guidelines: LDL-C < 1.8 mmol/L and <1.4 mmol/L,^35^ HbA1c < 7%,^30^ fasting plasma glucose < 7.0 mmol/L,^36^ and systolic BP < 130 mmHg and <140 mmHg.^37^

Missing data were primarily handled by complete-case analysis, excluding participants with missing values for weight change, outcomes, or covariates. The proportion of missing covariate data was limited (*n* = 115, corresponding to 7.8% of participants in adjusted models). Multiple imputation by chained equations was performed as a sensitivity analysis for missing baseline covariate values, missing 1-year weight values, and both combined. Missing values were imputed jointly, using predictive mean matching for continuous variables and logistic regression for binary variables. Twenty imputed datasets were generated. Cox models were fitted within each imputed dataset and estimates were pooled using Rubin’s rules.

The imputed datasets were used to evaluate the adjusted risk of clinical outcomes per 1% and 5% weight loss, as well as subgroup analyses for the primary outcome, 4-point MACE. Multiple imputation diagnostics included visual comparison of the distribution of 1-year weight change across complete-case participants, participants with imputed covariates only, participants with imputed weight values, and participants included in the full multiple imputation dataset. These analyses are presented in the Supplementary material.

A *P*-value lower than 0.05 was considered statistically significant. All analyses were conducted in R (version 4.4.2).

## Results

### Baseline characteristics

Among the 2447 overweight or obese patients enrolled in ELIPS with a planned 5-year follow-up, 490 (20.0%) were excluded by the 1-year visit due to death (*n* = 42), withdrawal (*n* = 55), loss to follow-up (*n* = 66), missing 1-year weight measurements (*n* = 323), or implausible weight values (*n* = 4). By the 5-year visit, an additional 484 participants (19.8%) were not analysed, including 293 lost to follow-up, 187 who withdrew, and 4 deaths with unknown dates. Overall, 1473 participants were included in the present analysis. The participant flowchart is shown in Supplementary material online, *Figure S1*.

The median weight change was 0%, and 47.5% of participants lost weight during the first year (*Table 1*). At baseline, patients in the weight loss group were older (62.9 ± 11.3 vs. 60.2 ± 11.5 years) and more obese (33.4% vs. 26.5%), had a slightly higher BMI [28.4 (26.5–31.0) vs. 27.8 (26.3–30.1) kg/m^2^] and lower renal function (80.8 ± 20.5 vs. 84.9 ± 20.7), and were less likely to be employed full-time (39.0% vs. 47.2%). The prevalence of smoking was substantially higher in the weight gain group (44.2% vs. 28.6%). Other clinical characteristics, including diabetes, ACS type, left ventricular ejection fraction, and medication use, were balanced between groups, except for diuretic (18.9% vs. 14.6%) and participation in cardiac rehabilitation after discharge, which was more frequent in the weight loss group (76.7% vs. 68.5%).

**Table 1:** Baseline characteristics of overweight and obese patients post-acute coronary syndrome

Category	Variable	All (*n* = 1473)	Weight loss^a^ (*n* = 700)	Weight gain or stable weight^a^ (*n* = 773)	*P*-value^b^
Demographics	Age (years)	61.5 ± 11.5	62.9 ± 11.3	60.2 ± 11.5	<0.01
	Female	223 (15.1%)	107 (15.3%)	116 (15.0%)	0.94
	Caucasian	1416 (96.4%)	680 (97.4%)	736 (95.5%)	0.06
	BMI (kg/m^2^)^c^	28.1 [26.3–30.5]	28.4 [26.5–31.0]	27.8 [26.3–30.1]	<0.01
	Obesity	439 (29.8%)	234 (33.4%)	205 (26.5%)	0.01
	Married or in partnership	1008 (68.5%)	488 (69.7%)	520 (67.4%)	0.38
	Living alone	308 (21.0%)	146 (20.9%)	162 (21.0%)	1.00
Education					0.32
University graduation (including applied sciences)		269 (18.7%)	131 (19.3%)	138 (18.2%)	
High school graduation		204 (14.2%)	106 (15.6%)	98 (12.9%)	
Apprenticeship or vocational school		704 (49.0%)	328 (48.3%)	376 (49.5%)	
Lower than apprenticeship or vocational school		261 (18.2%)	114 (16.8%)	147 (19.4%)	
Employment					<0.01
Full-time work		634 (43.3%)	273 (39.0%)	361 (47.2%)	
Part-time work		140 (9.6%)	63 (9.0%)	77 (10.1%)	
No employment/retired		691 (47.2%)	364 (52.0%)	327 (42.7%)	
Current smoking		542 (36.8%)	200 (28.6%)	342 (44.2%)	<0.01
Alcohol					0.20
Never in the past year		180 (12.4%)	73 (10.6%)	107 (14.1%)	
1–3 times a month		344 (23.8%)	161 (23.4%)	183 (24.1%)	
1–4 times a week		493 (34.1%)	244 (35.5%)	249 (32.8%)	
Every day or nearly every day		429 (29.7%)	209 (30.4%)	220 (29.0%)	
Blood pressure	Hypertension	820 (55.7%)	392 (56.0%)	428 (55.4%)	0.85
	Diastolic BP (mmHg)	76.0 ± 14.6	76.3 ± 14.5	75.8 ± 14.8	0.44
	Systolic BP (mmHg)	127.9 ± 23.5	128.5 ± 23.1	127.4 ± 23.9	0.34
	Heart rate (b.p.m.)	76.8 ± 16.0	77.4 ± 16.3	76.4 ± 15.8	0.23
Lipids	Cholesterol (mmol/L)	5.1 ± 1.2	5.1 ± 1.2	5.2 ± 1.2	0.25
	HDL (mmol/L)	1.1 ± 0.3	1.1 ± 0.3	1.1 ± 0.3	0.16
	LDL (mmol/L)	3.2 ± 1.1	3.2 ± 1.1	3.3 ± 1.1	0.54
	Triglyceride (mmol/L)	1.34 [0.9–2.06]	1.3 [0.9–2.0]	1.4 [0.9–2.1]	0.2
Diabetes	Diabetes	266 (18.1%)	127 (18.1%)	139 (18.0%)	0.99
	Antidiabetics at discharge	199 (13.5%)	98 (14.0%)	101 (13.1%)	0.65
	Insulin at discharge	99 (6.7%)	43 (6.2%)	56 (7.2%)	0.46
Renal function	eGFR (mL/min/1.73 m^2^)	83.0 ± 20.7	80.8 ± 20.5	84.9 ± 20.7	<0.01
Cardiovascular history	History of MI	225 (15.3%)	94 (13.4%)	131 (17.0%)	0.07
	History of PCI	244 (16.6%)	108 (15.4%)	136 (17.6%)	0.29
	History of CABG	59 (4.0%)	25 (3.6%)	34 (4.4%)	0.50
Killip class					0.57
	Killip I	1316 (90.4%)	631 (90.9%)	685 (89.9%)	
	Killip II–IV	140 (9.6%)	63 (9.1%)	77 (10.1%)	
LVEF at discharge (%)		52.8 ± 10.9	52.9 ± 11.0	52.7 ± 10.9	0.74
ACS type					0.20
NSTEMI		620 (42.1%)	293 (41.9%)	327 (42.3%)	
STEMI		781 (53.0%)	380 (54.3%)	401 (51.9%)	
UA		72 (4.9%)	27 (3.9%)	45 (5.8%)	
Medication at discharge	Aspirin	1466 (99.5%)	698 (99.7%)	768 (99.4%)	0.53
	Statin	1447 (98.2%)	688 (98.3%)	759 (98.2%)	1
	Beta-blocker	1246 (84.6%)	593 (84.7%)	653 (84.5%)	0.96
	Calcium blocker	93 (6.3%)	54 (7.7%)	39 (5.0%)	0.05
	Diuretic	245 (16.6%)	132 (18.9%)	113 (14.6%)	0.04
	PPI	387 (26.3%)	201 (28.7%)	186 (24.1%)	0.05
	RAASI	1360 (92.3%)	657 (93.9%)	703 (90.9%)	0.05
Cardiac rehabilitation after discharge		1049 (72.4%)	529 (76.7%)	520 (68.5%)	<0.01

BMI, body mass index; BP, blood pressure; HDL, high-density lipoprotein; LDL, low-density lipoprotein; eGFR, estimated glomerular filtration rate; MI, myocardial infarction; PCI, percutaneous coronary intervention; CABG, coronary artery bypass graft surgery; LVEF, left ventricular ejection fraction; ACS, acute coronary syndrome; STEMI, ST-elevation myocardial infarction; NSTEMI, non-ST-elevation myocardial infarction; UA, unstable angina; PPI, proton-pump inhibitors; RAASI, renin-angiotensin-aldosterone system inhibitors.

^a^Weight loss is defined as a weight change of <0% and weight gain or stable weight as ≥0%.

^b^One-way ANOVA for continuous variables expressed as mean ± SD and Kruskal–Wallis for continuous variables expressed as median [IQR] and χ² tests for categorical variables. Analyses excluded participants with missing data.

^c^Expressed as median [IQR].

Among participants who lost weight (*n* = 700), event rates were 19.4% for 4-point MACE, 13.1% for 3-point MACE, and 9.9% for all-cause death. In those who gained weight (*n* = 773), the corresponding event rates were 21.7% for 4-point MACE, 13.1% for 3-point MACE, and 8.9% for all-cause death.

The characteristics of the participants excluded for missing 1-year weight measurements are presented in Supplementary material online, *Table S1*. Compared with participants with available weight data, those with missing weight were more frequently female and had a higher prevalence of diabetes, a lower eGFR, and lower participation in cardiac rehabilitation.

Among the 916 participants with available 5-year weight measurements, participants in the weight gain group experienced a mean weight increase of 4.4% ± 4.6% at 1 year, which remained elevated at 5 years (3.8% ± 10.7%). In contrast, participants in the weight loss group showed a mean weight reduction of −5.2% ± 4.5% at 1 year and maintained a lower body weight at 5 years (−3.1% ± 7.6%). Weight trajectories are presented in Supplementary material online, *Figure S2*.

### Predictors of weight change

In our multivariate model (*Table 2*), older age, higher baseline BMI, and participation in cardiac rehabilitation after discharge were associated with weight loss (b per 10 years = −0.64; *P* < 0.01; b per 5 kg/m^2^ = −0.82; *P* < 0.001 and b = 1.75; *P* < 0.001, respectively). Smoking at baseline was strongly associated with weight gain (b = 2.61; *P* < 0.001). In exploratory analyses, changes in smoking behaviour over the first year were also associated with weight gain, with both smoking continuation and smoking cessation showing higher weight gain compared with persistent non-smoking (b = 1.46; *P* = 0.002 and b = 3.84; *P* < 0.001; Supplementary material online, *Table S2*). These findings should be interpreted cautiously, as changes in smoking status and weight occurred over the same time period, precluding clear temporal interpretation.

**Table 2:** Predictors of first-year post-acute coronary syndrome weight change (multivariable linear regression, *n* = 1291)

Variable	Reference	b (95% CI)	*P*-value
(Intercept)	–	9.24 (3.97–14.5)	<0.001
Sex	Female	0.46 (−0.54–1.46)	0.37
Age per 10 years	–	−0.64 (−1.06 to −0.23)	<0.01
Baseline BMI per 5 units (kg/m^2^)	–	−0.82 (−1.3 to −0.35)	<0.001
Education	–		0.18^a^
High school graduation	University graduation	−1.34 (−2.49 to −0.19)	0.02
Apprenticeship or vocational school	University graduation	−0.08 (−0.98–0.82)	0.86
Lower than apprenticeship or vocational school	University graduation	0.43 (−0.69–1.54)	0.45
Baseline smoking	Current smoking	2.61 (1.85–3.36)	<0.001
Alcohol consumption			0.28^a^
1–3 times a month	Never in the past year	−0.62 (−1.79–0.55)	0.30
1–4 times a week	Never in the past year	−1.12 (−2.25–0.02)	0.05
Every day or nearly every day	Never in the past year	−0.6 (−1.77–0.57)	0.31
Baseline total cholesterol per 1 mmol/L increase	–	−0.02 (−0.3–0.27)	0.91
Baseline eGFR per 1 mL/min/1.73 m^2^ increase	–	0 (−0.02–0.02)	0.64
Baseline diabetes	Presence	0.04 (−0.86–0.95)	0.93
Baseline hypertension	Presence	−0.01 (−0.74–0.71)	0.97
ACS type^b^	STEMI	0.27 (−0.41–0.96)	0.43
Cardiac rehabilitation after discharge	Participation	−1.75 (−2.54 to −0.96)	<0.001

One hundred and eighty-three participants were excluded due to missing values in adjustment covariates. Summary of missing: education: 35, smoking: 1, alcohol: 27, cholesterol: 93, eGFR: 37, cardiac rehabilitation: 24. Some participants had multiple missing. Positive coefficients indicate association with weight gain in percentage; negative coefficients indicate association with weight loss in percentage.

BMI, body mass index; eGFR, estimated glomerular filtration rate; STEMI, ST-elevation myocardial infarction.

^a^Linear *P*-value with education and alcohol consumption converted to a continuous variable.

^b^Compared to non-ST-elevation myocardial infarction or unstable angina.

### Association between weight change and outcome

In adjusted Cox models (*Table 3*), increased weight loss was associated with a reduction in 4-point MACE over 5 years (HR per 5% decrease = 0.90; 95% CI: 0.82–1.00; *P* = 0.04). A similar pattern was observed for 3-point MACE (HR = 0.90; 95% CI: 0.79–1.01; *P* = 0.08). No significant associations were found between weight change and all-cause or cardiovascular mortality (HR = 1.06; 95% CI: 0.91–1.24; *P* = 0.47). Similar results were obtained after adjustment for participation in cardiac rehabilitation or imputation of missing covariates and 1-year weight values (see Supplementary material online, *Tables S3–S6*). The distribution of 1-year weight change was similar across complete-case participants and the imputed datasets, supporting the plausibility of the imputed values (see Supplementary material online, *Figure S3*).

**Table 3:** Unadjusted and adjusted risk of clinical outcomes per 1% and 5% weight loss (*n* = 1358)

Outcome	Events	Model	HR per 1% WL with 95% CI	HR per 5% WL with 95% CI	*P*-value
4-point MACE	278	Unadjusted	0.99 (0.97–1.01)	0.95 (0.86–1.04)	0.23
		Adjusted	0.98 (0.96–1.00)	0.90 (0.82–1.00)	0.04
3-point MACE	178	Unadjusted	0.99 (0.97–1.01)	0.94 (0.84–1.05)	0.27
		Adjusted	0.98 (0.95–1.00)	0.90 (0.79–1.01)	0.08
All-cause death	116	Unadjusted	1.02 (0.99–1.05)	1.09 (0.95–1.26)	0.22
		Adjusted	1.01 (0.98–1.04)	1.06 (0.91–1.24)	0.47
Cardiovascular death	68	Unadjusted	1.01 (0.97–1.05)	1.06 (0.88–1.27)	0.55
		Adjusted	1.01 (0.97–1.05)	1.03 (0.84–1.26)	0.75
Myocardial infarction	93	Unadjusted	0.98 (0.95–1.01)	0.90 (0.77–1.05)	0.18
		Adjusted	0.97 (0.94–1.01)	0.88 (0.74–1.04)	0.13
Stroke	26	Unadjusted	0.96 (0.91–1.02)	0.82 (0.62–1.09)	0.17
		Adjusted	0.95 (0.89–1.01)	0.77 (0.57–1.04)	0.09
Revascularization	192	Unadjusted	0.99 (0.97–1.01)	0.94 (0.84–1.04)	0.24
		Adjusted	0.98 (0.96–1.00)	0.90 (0.80–1.01)	0.08

Adjusted for covariates: age, sex, BMI, smoking, total cholesterol, hypertension, diabetes, and estimated glomerular filtration rate. One hundred and fifteen participants were excluded due to missing values in adjustment covariates. Summary of missing: smoking: 1, cholesterol: 93, eGFR: 37. Some participants had multiple missing.

WL, weight loss; MACE, major adverse cardiovascular event.

**Figure 1:**
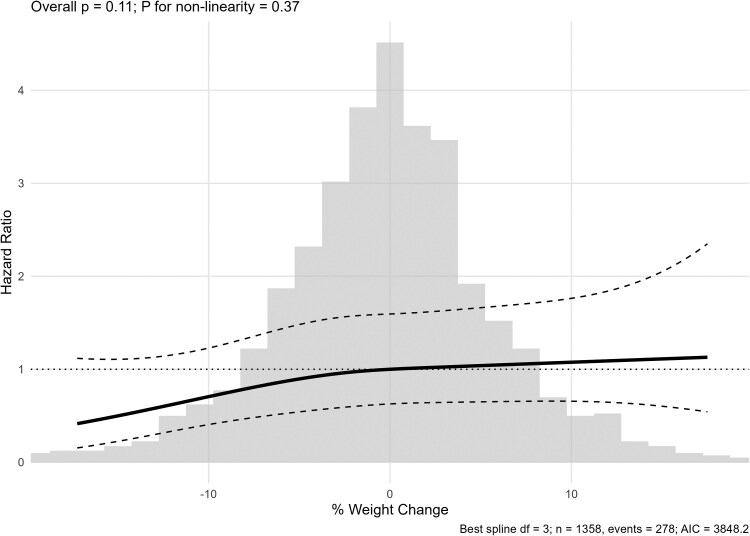
Adjusted hazard ratios for 4-point major adverse cardiovascular events according to per cent weight change during the first year after acute coronary syndrome. Curves represent adjusted hazard ratios estimated from Cox proportional hazards models using restricted cubic splines for per cent weight change. Hazard ratios are shown relative to 0% weight change, which serves as the reference value (hazard ratio = 1). The spline degrees of freedom = 3. The solid line represents the adjusted hazard ratio, the dashed lines represent 95% confidence intervals, and histograms illustrate the distribution of weight change in the study population. Negative values represent weight loss; positive values represent weight gain. All models were adjusted for age, sex, baseline body mass index, smoking status, total cholesterol, hypertension, diabetes, and estimated glomerular filtration rate. Overall *P*-value corresponds to the global significance of the spline term, and *P* for non-linearity reflects the comparison between spline and linear models. *N* = 1358, 115 participants were excluded due to missing values in adjustment covariates. Summary of missing: smoking: 1, cholesterol: 93, eGFR: 37. Some participants had multiple missing.

Adjusted HRs were modelled using restricted cubic splines to assess the association between weight change and clinical outcomes. For 4-point MACE (*Figure 1*), the HR was below 1 for weight loss and increased with weight gain, although CIs overlapped across the entire range of weight change. A similar pattern was observed for 3-point MACE (see Supplementary material online, *Figure S4*). For all-cause mortality (see Supplementary material online, *Figure S5*), the curve was flatter but showed a rise at greater weight loss and a decrease at greater weight gain. Analyses of individual MACE components (see Supplementary material online, *Figures S6*–*S9*) showed matching patterns. Coronary revascularization, myocardial infarction, and stroke showed lower risk with weight loss and increased risk with weight gain but with large CIs. For cardiovascular death, the spline curve was imprecise and did not show a clear association across the range of weight change. Results from Poisson regression models were consistent with those obtained from the Cox proportional hazards models (see Supplementary material online, *Figures S10*–*S12*).

### Subgroup analyses

Subgroup analyses for 4-point MACE (*Table 4*) showed consistent associations across subgroups, with no evidence of interaction (all *P* for interaction > 0.05). These findings were comparable after imputation of missing covariates (see Supplementary material online, *Table S7*). Similar results were observed for 3-point MACE (see Supplementary material online, *Table S8*). Weight loss was not associated with all-cause mortality across subgroups (see Supplementary material online, *Table S9*).

**Table 4:** Subgroup analysis of adjusted risk of 4-point major adverse cardiovascular events per 1% and 5% weight loss

Subgroup	Level	Events	*n*	HR per 1% WL (95% CI)	HR per 5% WL (95% CI)	Interaction *P*
Sex						0.55
	Male	239	1155	0.98 (0.96–1.00)	0.89 (0.80–0.99)	
	Female	39	203	0.99 (0.95–1.04)	0.98 (0.78–1.22)	
Age group						0.88
	<65 years	140	839	0.98 (0.96–1.01)	0.91 (0.81–1.03)	
	≥65 years	138	519	0.98 (0.95–1.01)	0.92 (0.78–1.07)	
BMI group						0.16
	<27 kg/m^2^	82	461	1.00 (0.97–1.04)	1.01 (0.85–1.20)	
	≥27 kg/m^2^	196	897	0.97 (0.95–0.99)	0.87 (0.77–0.97)	
Current smoking						0.74
	Yes	189	856	0.99 (0.96–1.01)	0.93 (0.82–1.05)	
	No	89	502	0.98 (0.95–1.02)	0.93 (0.79–1.09)	
ACS type						0.19
	STEMI	129	724	0.97 (0.94–1.00)	0.86 (0.75–0.99)	
	NSTEMI/UA	149	634	0.99 (0.96–1.02)	0.95 (0.83–1.09)	
Cardiac rehabilitation at discharge						0.30
	Yes	158	969	0.98 (0.96–1.01)	0.91 (0.80–1.03)	
	No	113	366	1.00 (0.96–1.03)	0.99 (0.83–1.18)	

Results from stratified and interaction analyses; *P*-values for interaction derived from likelihood ratio tests. Adjusted for age, sex, BMI, smoking status, cholesterol, hypertension, diabetes, and eGFR, excluding the stratification variable. One hundred and fifteen participants were excluded due to missing values in adjustment covariates. Summary of missing: smoking: 1, cholesterol: 93, eGFR: 37. Some participants had multiple missing.

BMI, body mass index; ACS, acute coronary syndrome; STEMI, ST-elevation myocardial infarction; NSTEMI, non-ST-elevation myocardial infarction; UA, unstable angina; *n*, number of participants; HR, hazard ratio; WL, weight loss; CI, confidence interval.

Unadjusted analyses are presented in Supplementary material online, *Tables S10–S12*. Weight loss was not associated with 4-point or 3-point MACE across subgroups. In unadjusted analyses only, weight loss showed a significant association with higher all-cause mortality among current smokers (unadjusted HR per 5% decrease = 1.28; 95% CI: 1.00–1.64; *P* = 0.05).

### Weight loss and achievement of 1-year cardiometabolic targets

At 1-year, weight loss was significantly associated with higher odds of achieving several cardiometabolic targets (see Supplementary material online, *Table S13*). In adjusted analyses, weight loss was also associated with the achievement of LDL-C targets: for LDL < 1.8 mmol/L, adjusted OR per 5% loss was 1.23 (95% CI 1.11–1.36; *P* < 0.001). For LDL < 1.4 mmol/L, OR was 1.15 (95% CI 1.01–1.30; *P* = 0.04). For systolic BP, weight loss was associated with both targets: for systolic BP < 130 mmHg, OR per 5% loss = 1.21 (95% CI 1.10–1.34; *P* < 0.001), and for systolic BP < 140 mmHg, OR per 5% loss = 1.24 (95% CI 1.11–1.39; *P* < 0.001). Similarly, for fasting glucose, a 5% weight loss was associated with increased OR of achieving glucose < 7 mmol/L [adjusted OR = 1.32 (95% CI 1.12–1.55; *P* < 0.001].

## Discussion

### Main findings

In this prospective Swiss cohort, weight loss during the first year after ACS was associated with a lower risk of subsequent MACE in patients with overweight or obesity at baseline. The lower risk of cardiovascular outcomes associated with weight reduction was accompanied by improved control of several cardiovascular risk factors at 1 year.

Each 5% relative reduction in body weight was associated with an ∼10% lower risk of MACE, an effect size within the range reported for recommended pharmacological therapies and recent GLP-1 receptor agonist trials in high–cardiovascular risk populations.^10,11,38–41^ Our findings reinforce the importance of weight management and the need to consider weight reduction as a treatment goal and quality indicator similar to targets already well-defined for LDL-C or BP.

Greater weight loss was associated with improved control of systolic BP, LDL-C, and fasting glucose, suggesting that the observed association between weight reduction and lower cardiovascular risk may be partly mediated by risk factor improvement. Regarding hypertension, this pathway is supported by a recent meta-analysis showing that incretin-based therapies lead to modest but clinically meaningful reductions in BP and lower all-cause mortality in adults with overweight or obesity, with greater weight loss associated with greater reductions in both systolic and diastolic BP.^42^ However, our study was not designed to formally test mediation.

As illustrated in the spline plot, the HR declined progressively with increasing weight loss, with no evidence of a distinct threshold indicating either a minimal or maximal amount of weight change required for benefit. Subgroup findings revealed some evidence for stronger associations in men and those with higher BMI at baseline, but these findings are exploratory and hypothesis-generating and should be interpreted cautiously given the limited power. The association observed for 4-point MACE was not driven solely by coronary revascularization. Effect estimates for revascularization were not greater than those observed for the composite MACE outcomes, and results for the 3-point MACE definition were directionally consistent, supporting a broader effect on hard cardiovascular outcomes.

This study was not designed to evaluate the reason (intentional or unintentional weight loss) or the treatment causing weight reduction. However, attendance at a cardiac rehabilitation programme after discharge was a strong predictor of weight reduction. Cardiac rehabilitation programmes are strongly supported by guidelines as an effective intervention to help patients to adopt a healthy lifestyle, improve confidence, and better self-management.^43^

By focusing on weight change during the first year, our approach aimed to capture early behavioural or therapeutic effects while limiting reverse causality because outcomes were only counted after Year 1. Nevertheless, weight change may still reflect broader lifestyle behaviours, environmental context, or medication adherence, and the associations observed should not be interpreted as causal. The use of GLP-1 RA was not specifically collected but their use was uncommon at the time of this study recruitment. Few studies have directly examined how weight change itself influences cardiovascular outcomes, whereas most literature focuses on controlling intermediate risk factors (e.g. BP, LDL-C).^44,45^ In this context, recent analyses from the SELECT trial highlight that GLP-1 RA reduce MACE largely independently of weight loss, suggesting cardioprotective mechanisms beyond adiposity alone.^46^ Therefore, the relationship between weight reduction and cardiovascular benefit may not be fully explained by GLP-1 RA-mediated weight loss alone. Biologically, the effect of weight reduction on inflammation, glycaemic control, lipid levels, and BP levels is already well-established, but the potential relation with cardiovascular outcomes after an ACS has been rarely reported.^47–51^ Our observational data represent a unique set of evidence emphasizing the clinical importance of weight reduction after ACS, especially since a clinical trial randomizing patients to lose weight or not is not feasible.

Despite the benefit of weight loss for cardiovascular health, the proportion of patients who can reduce their weight by 5% or more is very low (less than 20%). Our observations are similar to the data reported in the EUROASPIRE IV–V findings where 19.5% can achieve the recommended goals of 5% or more weight reduction.^3^ These findings reinforce the need to regularly address the importance of weight reduction during consultations. More research is needed in this context to understand which types of behavioural interventions can have the most favourable impact on weight reduction and how cardiologists perceive their role and skills to promote weight reduction on top of all other treatment goals.

### Strengths and limitations

Key strengths include a well-defined, multicentre cohort with available data for weight and covariates, adjudicated outcomes, and robust modelling using spline and subgroup analyses. However, several limitations deserve consideration.

First, the study may have had limited power for some secondary outcomes due to the small number of events. Second, weight change was assessed only during the first year after ACS, and longer-term trajectories were not captured. Third, intentional vs. unintentional weight loss could not be distinguished, despite their differing prognostic implications. Unintentional weight loss after ACS may reflect underlying frailty or disease progression. Although follow-up was initiated at 1 year to reduce the risk of reverse causality, this possibility cannot be fully excluded. Fourth, residual confounding cannot be excluded, as information on several relevant lifestyle, behavioural, and clinical factors, including physical activity, dietary habits, medication adherence, and disease severity, was not available. Although additional adjustment for participation in cardiac rehabilitation yielded results consistent with the main findings, unmeasured or incompletely measured factors may still have influenced the observed associations. Fifth, measures of adiposity beyond BMI, such as waist circumference or indices of body fat distribution, were not available. These parameters may better capture central adiposity and cardiometabolic risk, and their absence may have limited the precision of obesity phenotyping in our analyses. Sixth, we did not apply a ‘stable weight’ reference category to avoid arbitrary thresholds, which lack consensus across the literature.^52,53^ Seventh, missing weight data may have introduced selection bias, likely reflecting differences in follow-up modality, with some participants followed by telephone rather than in-person visits, thereby precluding standardized weight measurements. Finally, detailed information on the intensity of lipid-lowering therapy and the use of newer cardioprotective agents, such as GLP-1 receptor agonists and SGLT2 inhibitors, was not available. Nevertheless, the biological relationship between weight change and cardiovascular risk remains clinically relevant. Future studies will help determine whether these findings extend to populations receiving contemporary, guideline-directed care.

To conclude, in overweight and obese patients after ACS, weight loss during the first year was associated with a lower 5-year risk of 4-point MACE. These findings support recommendations for structured weight management following ACS and sustained, individualized interventions to reduce long-term cardiovascular risk.

### Declaration of generative artificial intelligence (AI) in scientific writing

The authors used AI tools to refine, summarize, and shorten sections of the manuscript and approved the final version of the article. AI was also used for the *graphical abstract*.

## Supplementary Material

oeag105_Supplementary_Data

## Data Availability

The data that support the findings of this study are available from the corresponding author upon reasonable request.
