# Supplementary material for: Weight change and long-term cardiovascular outcomes in overweight and obese patients after acute coronary syndromes
Source: Eur Heart J Open. 2026 Jun 9;6(3):oeag105. doi: 10.1093/ehjopen/oeag105 (PMC13293091; doi:10.1093/ehjopen/oeag105)
Supplement: oeag105_Supplementary_Data [file oeag105_supplementary_data.docx]

**Supplemental materials**

**Weight Change and Cardiovascular Outcomes in Overweight and Obese Patients after Acute Coronary Syndromes**

Artels L. et al.

**Table of Contents**

[Table S1. Baseline characteristics of participants with and without available 1-year weight data among those with completed 5-year follow-up 2](#_Toc229488605)

[Table S2. Predictors of first-year post-ACS weight change (multivariable linear regression) with changes in smoking status (n = 1,286) 3](#_Toc229488606)

[Table S3. Unadjusted and adjusted risk of clinical outcomes per 1% and 5% weight loss adjusted for age, sex, BMI, smoking, total cholesterol, hypertension, diabetes, eGFR and cardiac rehabilitation 4](#_Toc229488607)

[Table S4. Unadjusted and adjusted risk of clinical outcomes per 1% and 5% weight loss with multiple imputation of missing covariates (n imputed = 115, n participants =1473) 5](#_Toc229488608)

[Table S5. Unadjusted and adjusted risk of clinical outcomes per 1% and 5% weight loss with multiple imputation of missing 1-year weight (n imputed = 213, n with missing covariates = 139, n participants = 1547) 6](#_Toc229488609)

[Table S6. Unadjusted and adjusted risk of clinical outcomes per 1% and 5% weight loss with multiple imputation of missing covariates and 1-year weight (n imputed = 328, n participants = 1686) 7](#_Toc229488610)

[Table S7. Subgroup analysis of adjusted risk of 4-point MACE per 1% and 5% weight loss with multiple imputation of missing covariates 8](#_Toc229488611)

[Table S8. Subgroup analysis of adjusted risk of 3-point MACE per 1% and 5% weight loss 9](#_Toc229488612)

[Table S9. Subgroup analysis of adjusted risk of all-cause death per 1% and 5% weight loss 10](#_Toc229488613)

[Table S10. Subgroup analysis of unadjusted risk of 4-point MACE per 1% and 5% weight loss 11](#_Toc229488614)

[Table S11. Subgroup analysis of unadjusted risk of 3-point MACE per 1% and 5% weight loss 12](#_Toc229488615)

[Table S12. Subgroup analysis of unadjusted risk of all-cause death per 1% and 5% weight loss 13](#_Toc229488616)

[Table S13. Achievement of secondary prevention cardiometabolic target per 1% and 5% weight loss 14](#_Toc229488617)

[Figure S1. Flowchart of Study Participants 16](#_Toc229488618)

[Figure S2. Weight Change Trajectories According to 1-year Weight Change Group 17](#_Toc229488619)

[Figure S3. Distribution of 1-year weight change across complete-case and multiple imputation datasets 18](#_Toc229488620)

[Figure S4. Adjusted Hazard Ratio for 3-point MACE by Percent Weight Change 19](#_Toc229488621)

[Figure S5. Adjusted Hazard Ratio for All-cause Death by Percent Weight Change 20](#_Toc229488622)

[Figure S6. Adjusted Hazard Ratio for Cardiovascular Death by Percent Weight Change 21](#_Toc229488623)

[Figure S7. Adjusted Hazard Ratio for Myocardial Infarction by Percent Weight Change 22](#_Toc229488624)

[Figure S8. Adjusted Hazard Ratio for Coronary Revascularization by Percent Weight Change 23](#_Toc229488625)

[Figure S9. Adjusted Hazard Ratio for Stroke by Percent Weight Change 24](#_Toc229488626)

[Figure S10. Adjusted Incidence Rate for 4-point MACE by Percent Weight Change 25](#_Toc229488627)

[Figure S11. Adjusted Incidence Rate for 3-point MACE by Percent Weight Change 26](#_Toc229488628)

[Figure S12. Adjusted Incidence Rate for All-cause Death by Percent Weight Change 27](#_Toc229488629)

**Table S1**. Baseline characteristics of participants with and without available 1-year weight data among those with completed 5-year follow-up

| Baseline characteristic | 1-year weight not measured^1^  (n = 213) | 1-year weight measured^1^  (n = 1’473) | p-value^2^ |
| --- | --- | --- | --- |
| Age | 63.4 ± 12.1 | 61.5 ± 11.5 | 0.03 |
| Female | 63 (30%) | 223 (15%) | <0.01 |
| Obesity | 75 (35%) | 439 (30%) | 0.13 |
| Current smoking | 85 (40%) | 542 (37%) | 0.43 |
| Diabetes | 51 (24%) | 266 (18%) | 0.04 |
| Hypertension | 133 (62%) | 820 (56%) | 0.06 |
| Cholesterol | 5.07 ± 1.23 | 5.14 ± 1.23 | 0.46 |
| eGFR | 79.4 ± 21.4 | 83.0 ± 20.7 | 0.03 |
| ACS type |  |  | 0.22 |
| NSTEMI | 100 (47%) | 620 (42%) |  |
| STEMI | 107 (50%) | 781 (53%) |  |
| UA | 6 (2.8%) | 72 (4.9%) |  |
| Cardiac Rehabilitation | 113 (62%) | 1,049 (72%) | <0.01 |
| Comorbidity |  |  | 0.09 |
| None | 27 (13%) | 214 (15%) |  |
| 1-2 | 170 (80%) | 1,197 (81%) |  |
| ≥3 | 16 (7%) | 62 (4%) |  |
| ^1^n (%); mean ± SD | | | |
| ^2^Pearson's Chi-squared test; Wilcoxon rank sum test; Fisher's exact test | | | |

Table S2. Predictors of first-year post-ACS weight change (multivariable linear regression) with changes in smoking status (n = 1,286)

| Variable | Comparison/Reference | b (95% CI) | p-value |
| --- | --- | --- | --- |
| (Intercept) | - | 9.59 (4.3–14.89) | <0.001 |
| Sex | Female | 0.52 (-0.47–1.52) | 0.301 |
| Age per 10 years | - | -0.62 (-1.04–-0.21) | 0.003 |
| Baseline BMI per 5 units (kg/m^2^) | - | -0.8 (-1.27–-0.32) | 0.001 |
| Education | - |  |  |
| High school graduation | University graduation | -1.38 (-2.53–-0.23) | 0.018 |
| Apprenticeship or vocational school | University graduation | -0.11 (-1.01–0.78) | 0.804 |
| Lower than apprenticeship or vocational school | University graduation | 0.43 (-0.68–1.54) | 0.443 |
| Smoking status change |  |  |  |
| Started smoking | Continued not smoking | 1.98 (-1.65–5.6) | 0.285 |
| Stopped smoking | Continued not smoking | 3.84 (2.91–4.78) | <0.001 |
| Continued smoking | Continued not smoking | 1.46 (0.53–2.38) | 0.002 |
| Alcohol Consumption |  |  |  |
| 1-3 times a month | Never in the past year | -0.66 (-1.83–0.51) | 0.267 |
| 1-4 times a week | Never in the past year | -1.17 (-2.3–-0.04) | 0.042 |
| Every day or nearly every day | Never in the past year | -0.66 (-1.82–0.51) | 0.268 |
| Baseline total cholesterol per 1 mmol/L Increase | - | -0.06 (-0.34–0.22) | 0.677 |
| Baseline eGFR per 1 mL/min/1.73 m^2^ Increase | - | 0 (-0.02–0.02) | 0.629 |
| Baseline diabetes | Presence | -0.02 (-0.92–0.88) | 0.964 |
| Baseline hypertension | Presence | -0.04 (-0.76–0.68) | 0.904 |
| ACS type^1^ | STEMI | -0.27 (-0.95–0.41) | 0.437 |
| Cardiac rehabilitation after discharge | Participation | -1.9 (-2.69–-1.11) | <0.001 |

BMI: body mass index, eGFR: estimated glomerular filtration rate, STEMI: ST-elevation myocardial infarction.

187 participants were excluded due to missing values in adjustment covariates. Summary of missing: education: 35, smoking evolution: 4, alcohol: 27, cholesterol: 93, eGFR: 37, cardiac rehabilitation: 24. Some participants had multiple missing. Positive coefficients indicate association with weight gain in percentage; negative coefficients indicate association with weight loss in percentage.

1: compared to non-ST-elevation myocardial infarction or unstable angina

Table S3. Unadjusted and adjusted risk of clinical outcomes per 1% and 5% weight loss adjusted for age, sex, BMI, smoking, total cholesterol, hypertension, diabetes, eGFR and cardiac rehabilitation

| Outcome | Events | Model | HR per 1% WL with 95% CI | HR per 5% WL with 95% CI | p-value |
| --- | --- | --- | --- | --- | --- |
| 4-point MACE | 271 | Unadjusted | 0.990 (0.97–1.01) | 0.951 (0.87–1.04) | 0.29 |
|  |  | Adjusted | 0.987 (0.97–1.01) | 0.936 (0.85–1.04) | 0.202 |
| 3-point MACE | 172 | Unadjusted | 0.989 (0.97–1.01) | 0.946 (0.84–1.06) | 0.353 |
|  |  | Adjusted | 0.987 (0.96–1.01) | 0.935 (0.82–1.06) | 0.307 |
| All-cause death | 112 | Unadjusted | 1.022 (0.99–1.05) | 1.117 (0.97–1.29) | 0.13 |
|  |  | Adjusted | 1.027 (0.99–1.06) | 1.145 (0.97–1.35) | 0.103 |
| Cardiovascular death | 65 | Unadjusted | 1.012 (0.97–1.05) | 1.062 (0.88–1.28) | 0.532 |
|  |  | Adjusted | 1.024 (0.98–1.07) | 1.127 (0.91–1.40) | 0.279 |
| Myocardial infarction | 91 | Unadjusted | 0.980 (0.95–1.01) | 0.903 (0.77–1.06) | 0.208 |
|  |  | Adjusted | 0.980 (0.95–1.01) | 0.905 (0.76–1.08) | 0.258 |
| Stroke | 24 | Unadjusted | 0.972 (0.91–1.03) | 0.868 (0.64–1.18) | 0.363 |
|  |  | Adjusted | 0.966 (0.90–1.03) | 0.840 (0.60–1.17) | 0.306 |
| Revascularization | 189 | Unadjusted | 0.987 (0.97–1.01) | 0.938 (0.84–1.05) | 0.259 |
|  |  | Adjusted | 0.984 (0.96–1.01) | 0.923 (0.82–1.04) | 0.191 |

WL: weight loss. 4-point MACE: cardiovascular death, non-fatal myocardial infarction, stroke or coronary revascularization. 3-point MACE: cardiovascular death, non-fatal myocardial infarction or stroke.

Table S4. Unadjusted and adjusted risk of clinical outcomes per 1% and 5% weight loss with multiple imputation of missing covariates (n imputed = 115, n participants = 1’473)

| Outcome | Events | Model | HR per 1% WL with 95% CI | HR per 5% WL with 95% CI | p-value |
| --- | --- | --- | --- | --- | --- |
| 4-point MACE | 299 | Unadjusted | 0.99 (0.97–1.01) | 0.96 (0.88–1.05) | 0.36 |
|  |  | Adjusted | 0.98 (0.96–1.00) | 0.91 (0.83–1.00) | 0.05 |
| 3-point MACE | 194 | Unadjusted | 0.99 (0.97–1.01) | 0.96 (0.86–1.07) | 0.44 |
|  |  | Adjusted | 0.98 (0.96–1.00) | 0.91 (0.80–1.02) | 0.11 |
| All-cause death | 129 | Unadjusted | 1.02 (0.99–1.05) | 1.09 (0.96–1.25) | 0.19 |
|  |  | Adjusted | 1.01 (0.98–1.04) | 1.04 (0.89–1.20) | 0.65 |
| Cardiovascular death | 75 | Unadjusted | 1.01 (0.98–1.05) | 1.06 (0.89–1.27) | 0.51 |
|  |  | Adjusted | 1.00 (0.96–1.04) | 1.00 (0.83–1.22) | 0.98 |
| Myocardial infarction | 97 | Unadjusted | 0.99 (0.95–1.02) | 0.93 (0.79–1.08) | 0.33 |
|  |  | Adjusted | 0.98 (0.95–1.01) | 0.91 (0.77–1.07) | 0.25 |
| Stroke | 28 | Unadjusted | 0.96 (0.91–1.01) | 0.80 (0.61–1.06) | 0.12 |
|  |  | Adjusted | 0.94 (0.89–1.00) | 0.74 (0.55–1.00) | 0.05 |
| Revascularization | 203 | Unadjusted | 0.99 (0.97–1.01) | 0.96 (0.86–1.06) | 0.40 |
|  |  | Adjusted | 0.98 (0.96–1.01) | 0.92 (0.82–1.03) | 0.14 |

WL: weight loss. 4-point MACE: cardiovascular death, non-fatal myocardial infarction, stroke or coronary revascularization. 3-point MACE: cardiovascular death, non-fatal myocardial infarction or stroke.

Adjusted for covariates: age, sex, BMI, smoking, total cholesterol, hypertension, diabetes, and eGFR

Table S5. Unadjusted and adjusted risk of clinical outcomes per 1% and 5% weight loss with multiple imputation of missing 1-year weight (n imputed = 213, n with missing covariates = 139, n participants = 1’547)

| Outcome | Events | Model | HR per 1% WL with 95% CI | HR per 5% WL with 95% CI | p-value |
| --- | --- | --- | --- | --- | --- |
| 4-point MACE | 321 | Unadjusted | 0.99 (0.97–1.01) | 0.96 (0.88–1.05) | 0.38 |
|  |  | Adjusted | 0.98 (0.96–1.00) | 0.92 (0.84–1.01) | 0.08 |
| 3-point MACE | 210 | Unadjusted | 0.99 (0.97–1.02) | 0.97 (0.87–1.08) | 0.54 |
|  |  | Adjusted | 0.98 (0.96–1.01) | 0.92 (0.82–1.03) | 0.16 |
| All-cause death | 148 | Unadjusted | 1.02 (0.99–1.05) | 1.10 (0.96–1.26) | 0.16 |
|  |  | Adjusted | 1.01 (0.98–1.04) | 1.06 (0.92–1.23) | 0.43 |
| Cardiovascular death | 85 | Unadjusted | 1.02 (0.98–1.05) | 1.08 (0.90–1.29) | 0.44 |
|  |  | Adjusted | 1.01 (0.97–1.05) | 1.04 (0.86–1.27) | 0.68 |
| Myocardial infarction | 106 | Unadjusted | 0.98 (0.95–1.01) | 0.91 (0.78–1.06) | 0.23 |
|  |  | Adjusted | 0.98 (0.95–1.01) | 0.89 (0.75–1.05) | 0.16 |
| Stroke | 33 | Unadjusted | 0.97 (0.91–1.03) | 0.85 (0.64–1.15) | 0.30 |
|  |  | Adjusted | 0.96 (0.90–1.02) | 0.80 (0.57–1.10) | 0.19 |
| Revascularization | 214 | Unadjusted | 0.99 (0.97–1.01) | 0.94 (0.85–1.05) | 0.26 |
|  |  | Adjusted | 0.98 (0.96–1.00) | 0.91 (0.81–1.02) | 0.09 |

WL: weight loss. 4-point MACE: cardiovascular death, non-fatal myocardial infarction, stroke or coronary revascularization. 3-point MACE: cardiovascular death, non-fatal myocardial infarction or stroke.

Adjusted for covariates: age, sex, BMI, smoking, total cholesterol, hypertension, diabetes, and eGFR

Table S6. Unadjusted and adjusted risk of clinical outcomes per 1% and 5% weight loss with multiple imputation of missing covariates and 1-year weight (n imputed = 328, n participants = 1’686)

| Outcome | Events | Model | HR per 1% WL with 95% CI | HR per 5% WL with 95% CI | p-value |
| --- | --- | --- | --- | --- | --- |
| 4-point MACE | 349 | Unadjusted | 0.99 (0.98–1.01) | 0.97 (0.89–1.06) | 0.52 |
|  |  | Adjusted | 0.98 (0.97–1.00) | 0.92 (0.84–1.01) | 0.09 |
| 3-point MACE | 233 | Unadjusted | 1.00 (0.97–1.02) | 0.98 (0.88–1.09) | 0.69 |
|  |  | Adjusted | 0.98 (0.96–1.01) | 0.92 (0.82–1.04) | 0.18 |
| All-cause death | 167 | Unadjusted | 1.02 (0.99–1.04) | 1.09 (0.96–1.24) | 0.19 |
|  |  | Adjusted | 1.00 (0.98–1.03) | 1.02 (0.88–1.18) | 0.79 |
| Cardiovascular death | 97 | Unadjusted | 1.02 (0.98–1.05) | 1.08 (0.91–1.29) | 0.36 |
|  |  | Adjusted | 1.00 (0.97–1.04) | 1.02 (0.84–1.22) | 0.88 |
| Myocardial infarction | 112 | Unadjusted | 0.98 (0.95–1.01) | 0.92 (0.79–1.07) | 0.29 |
|  |  | Adjusted | 0.98 (0.95–1.01) | 0.90 (0.77–1.06) | 0.23 |
| Stroke | 37 | Unadjusted | 0.97 (0.92–1.02) | 0.85 (0.66–1.10) | 0.22 |
|  |  | Adjusted | 0.95 (0.90–1.01) | 0.79 (0.59–1.04) | 0.11 |
| Revascularization | 227 | Unadjusted | 0.99 (0.97–1.01) | 0.96 (0.87–1.07) | 0.47 |
|  |  | Adjusted | 0.99 (0.96–1.01) | 0.93 (0.83–1.04) | 0.20 |

WL: weight loss. 4-point MACE: cardiovascular death, non-fatal myocardial infarction, stroke or coronary revascularization. 3-point MACE: cardiovascular death, non-fatal myocardial infarction or stroke.

Adjusted for covariates: age, sex, BMI, smoking, total cholesterol, hypertension, diabetes, and eGFR.

Multiple imputation with predictive mean matching for continuous variables and logistic regression for binary variables (m = 20). Cox models were fitted within each imputed dataset and pooled using Rubin’s rules.

Table S7. Subgroup analysis of adjusted risk of 4-point MACE per 1% and 5% weight loss with multiple imputation of missing covariates

| Subgroup | |  | Events | n | HR per 1% (95% CI) | HR per 5% (95% CI) | | Interaction p |
| --- | --- | --- | --- | --- | --- | --- | --- | --- |
| Sex | |  |  |  |  | |  | 0.33 |
| Male | |  | 255 | 1,250 | 0.98 (0.96–1.00) | | 0.89 (0.80–0.98) |  |
| Female | |  | 44 | 223 | 1.00 (0.96–1.04) | | 1.00 (0.80–1.24) |  |
| Age group | |  |  |  |  | |  | 0.42 |
| <65 | |  | 148 | 901 | 0.98 (0.96–1.00) | | 0.90 (0.80–1.02) |  |
| ≥65 | |  | 151 | 572 | 0.99 (0.96–1.03) | | 0.97 (0.83–1.13) |  |
| BMI group | |  |  |  |  | |  | 0.12 |
| <27 | |  | 88 | 492 | 1.00 (0.97–1.04) | | 1.02 (0.86–1.22) |  |
| ≥27 | |  | 211 | 981 | 0.97 (0.95–0.99) | | 0.87 (0.77–0.97) |  |
| Current smoking | |  |  |  |  | |  | 0.76 |
| Yes | |  | 93 | 542 | 0.99 (0.96–1.02) | | 0.94 (0.80–1.10) |  |
| No | |  | 206 | 931 | 0.99 (0.96–1.01) | | 0.93 (0.83–1.05) |  |
| ACS type | |  |  |  |  | |  | 0.14 |
|  | STEMI |  | 136 | 781 | 0.97 (0.95–1.00) | | 0.86 (0.76–0.99) |  |
|  | NSTEMI / UA |  | 163 | 692 | 0.99 (0.97–1.02) | | 0.97 (0.85–1.10) |  |
|  | Cardiac rehabilitation after discharge | | |  |  | |  | 0.37 |
|  | Yes |  | 175 | 1,070 | 0.98 (0.96–1.01) | | 0.91 (0.81–1.03) |  |
|  | No |  | 124 | 403 | 0.99 (0.96–1.03) | | 0.97 (0.82–1.14) |  |

Results from stratified and interaction analyses; p-values for interaction derived from likelihood ratio tests.

Adjusted for age, sex, BMI, smoking status, cholesterol, hypertension, diabetes, and eGFR, excluding the stratification variable. BMI: body mass index, ACS: acute coronary syndrome, STEMI: ST-elevation myocardial infarction, NSTEMI: non-ST-elevation myocardial infarction, UA: unstable angina, n: number of participants, HR: hazard ratio; WL: weight loss; CI: confidence interval

Table S8. Subgroup analysis of adjusted risk of 3-point MACE per 1% and 5% weight loss

| Subgroup | | Events | n | HR per 1% (95% CI) | HR per 5% (95% CI) | Interaction p |
| --- | --- | --- | --- | --- | --- | --- |
| Sex | |  |  |  |  | 0.52 |
|  | Male | 151 | 1,155 | 0.98 (0.95–1.01) | 0.90 (0.79–1.03) |  |
|  | Female | 27 | 203 | 0.97 (0.93–1.02) | 0.88 (0.68–1.13) |  |
| Age group | |  |  |  |  | 0.94 |
|  | < 65 years | 79 | 839 | 0.98 (0.95–1.02) | 0.92 (0.78–1.08) |  |
|  | ≥65 years | 99 | 519 | 0.98 (0.94–1.02) | 0.90 (0.75–1.09) |  |
| BMI group | |  |  |  |  | 0.19 |
|  | < 27 kg/m^2^ | 54 | 461 | 1.00 (0.96–1.05) | 1.02 (0.82–1.27) |  |
|  | ≥ 27 kg/m^2^ | 124 | 897 | 0.97 (0.94–1.00) | 0.85 (0.73–0.98) |  |
| Current smoking | |  |  |  |  | 0.86 |
|  | Yes | 121 | 856 | 0.98 (0.95–1.01) | 0.91 (0.78–1.07) |  |
|  | No | 57 | 502 | 0.99 (0.95–1.03) | 0.97 (0.79–1.18) |  |
| ACS type |  |  |  |  |  | 0.29 |
|  | STEMI | 73 | 724 | 0.97 (0.93–1.00) | 0.85 (0.71–1.02) |  |
|  | NSTEMI / UA | 105 | 634 | 0.99 (0.96–1.02) | 0.95 (0.81–1.13) |  |
| Cardiac rehabilitation at discharge | |  |  |  |  | 0.89 |
|  | Yes | 93 | 969 | 0.99 (0.95–1.02) | 0.94 (0.79–1.11) |  |
|  | No | 79 | 366 | 0.98 (0.94–1.02) | 0.91 (0.74–1.13) |  |

Adjusted for age, sex, BMI, smoking status, cholesterol, hypertension, diabetes, and eGFR, excluding the stratification variable.

BMI: body mass index, ACS: acute coronary syndrome, STEMI: ST-elevation myocardial infarction, NSTEMI: non-ST-elevation myocardial infarction, UA: unstable angina.

115 participants were excluded due to missing values in adjustment covariates. Summary of missing: smoking: 1, cholesterol: 93, eGFR: 37. Some participants had multiple missing. Cardiac rehabilitation status, used only for subgroup analyses, had 24 missing values

**Table S9**. Subgroup analysis of adjusted risk of all-cause death per 1% and 5% weight loss

| Subgroup | | Events | n | HR per 1% (95% CI) | HR per 5% (95% CI) | Interaction p |
| --- | --- | --- | --- | --- | --- | --- |
| Sex | |  |  |  |  | 0.49 |
|  | Male | 92 | 1,155 | 1.02 (0.98–1.05) | 1.08 (0.91–1.29) |  |
|  | Female | 24 | 203 | 0.99 (0.93–1.05) | 0.94 (0.68–1.30) |  |
| Age group | |  |  |  |  | 0.14 |
|  | < 65 years | 33 | 839 | 1.03 (0.98–1.08) | 1.17 (0.92–1.48) |  |
|  | ≥ 65 years | 83 | 519 | 0.99 (0.95–1.03) | 0.96 (0.79–1.17) |  |
| BMI group | |  |  |  |  | 0.68 |
|  | < 27 kg/m^2^ | 46 | 461 | 1.02 (0.97–1.06) | 1.09 (0.87–1.36) |  |
|  | ≥ 27 kg/m^2^ | 70 | 897 | 1.01 (0.97–1.05) | 1.04 (0.84–1.28) |  |
| Current smoking | |  |  |  |  | 0.82 |
|  | Yes | 35 | 856 | 1.02 (0.97–1.07) | 1.09 (0.85–1.39) |  |
|  | No | 81 | 502 | 1.01 (0.97–1.05) | 1.06 (0.87–1.29) |  |
| ACS type |  |  |  |  |  | 0.98 |
|  | STEMI | 43 | 724 | 1.02 (0.97–1.06) | 1.08 (0.85–1.37) |  |
|  | NSTEMI / UA | 73 | 634 | 1.02 (0.98–1.06) | 1.10 (0.89–1.35) |  |
| Cardiac rehabilitation at discharge | |  |  |  |  | 0.29 |
|  | Yes | 51 | 969 | 1.00 (0.96–1.05) | 1.02 (0.81–1.29) |  |
|  | No | 61 | 366 | 1.04 (0.99–1.10) | 1.24 (0.97–1.58) |  |

Adjusted for age, sex, BMI, smoking status, cholesterol, hypertension, diabetes, and eGFR, excluding the stratification variable. BMI: body mass index, ACS: acute coronary syndrome, STEMI: ST-elevation myocardial infarction, NSTEMI: non-ST-elevation myocardial infarction, UA: unstable angina.

115 participants were excluded due to missing values in adjustment covariates. Summary of missing: smoking: 1, cholesterol: 93, eGFR: 37. Some participants had multiple missing. Cardiac rehabilitation status, used only for subgroup analyses, had 24 missing values

**Table S10.** Subgroup analysis of unadjusted risk of 4-point MACE per 1% and 5% weight loss

| Subgroup | | Events | n | HR per 1% (95% CI) | HR per 5% (95% CI) | Interaction p |
| --- | --- | --- | --- | --- | --- | --- |
| Sex | |  |  |  |  | 0.18 |
|  | Male | 255 | 1,250 | 0.99 (0.97–1.01) | 0.93 (0.84–1.03) |  |
|  | Female | 44 | 223 | 1.02 (0.98–1.06) | 1.09 (0.89–1.33) |  |
| Age group | |  |  |  |  | 0.86 |
|  | < 65 years | 148 | 901 | 0.98 (0.96–1.01) | 0.92 (0.82–1.04) |  |
|  | ≥ 65 years | 151 | 572 | 0.99 (0.96–1.02) | 0.94 (0.80–1.09) |  |
| BMI group | |  |  |  |  | 0.22 |
|  | < 27 kg/m^2^ | 88 | 492 | 1.01 (0.98–1.04) | 1.04 (0.89–1.22) |  |
|  | ≥ 27 kg/m^2^ | 211 | 981 | 0.98 (0.96–1.00) | 0.92 (0.83–1.02) |  |
| Current smoking | |  |  |  |  | 0.52 |
|  | Yes | 206 | 930 | 0.99 (0.96–1.02) | 0.97 (0.83–1.13) |  |
|  | No | 93 | 542 | 0.98 (0.96–1.00) | 0.91 (0.81–1.02) |  |
| ACS type |  |  |  |  |  | 0.09 |
|  | STEMI | 136 | 781 | 0.98 (0.95–1.00) | 0.90 (0.79–1.01) |  |
|  | NSTEMI / UA | 163 | 692 | 1.01 (0.98–1.03) | 1.04 (0.92–1.18) |  |
| Cardiac rehabilitation at discharge | |  |  |  |  | 0.70 |
|  | Yes | 170 | 1,049 | 1.00 (0.97–1.02) | 0.98 (0.87–1.09) |  |
|  | No | 122 | 400 | 1.00 (0.97–1.04) | 1.02 (0.86–1.19) |  |

BMI: body mass index, ACS: acute coronary syndrome, STEMI: ST-elevation myocardial infarction, NSTEMI: non-ST-elevation myocardial infarction, UA: unstable angina. Current smoking status had 1 missing value and cardiac rehabilitation status had 24 missing values

**Table S11.** Subgroup analysis of unadjusted risk of 3-point MACE per 1% and 5% weight loss

| Subgroup | | Events | n | HR per 1% (95% CI) | HR per 5% (95% CI) | Interaction p |
| --- | --- | --- | --- | --- | --- | --- |
| Sex | |  |  |  |  | 0.79 |
|  | Male | 163 | 1,250 | 0.99 (0.97–1.01) | 0.95 (0.84–1.08) |  |
|  | Female | 31 | 223 | 1.00 (0.95–1.04) | 0.99 (0.78–1.25) |  |
| Age group | |  |  |  |  | 0.88 |
|  | < 65 years | 84 | 901 | 0.98 (0.95–1.01) | 0.90 (0.77–1.05) |  |
|  | ≥ 65 years | 110 | 572 | 0.98 (0.95–1.02) | 0.92 (0.76–1.10) |  |
| BMI group | |  |  |  |  | 0.18 |
|  | < 27 kg/m^2^ | 58 | 492 | 1.01 (0.97–1.06) | 1.07 (0.88–1.31) |  |
|  | ≥ 27 kg/m^2^ | 136 | 981 | 0.98 (0.96–1.01) | 0.91 (0.80–1.03) |  |
| Current smoking | |  |  |  |  | 0.33 |
|  | Yes | 134 | 930 | 1.00 (0.96–1.04) | 1.00 (0.83–1.21) |  |
|  | No | 60 | 542 | 0.98 (0.95–1.01) | 0.89 (0.77–1.03) |  |
| ACS type |  |  |  |  |  | 0.19 |
|  | STEMI | 80 | 781 | 0.98 (0.95–1.01) | 0.89 (0.76–1.05) |  |
|  | NSTEMI / UA | 114 | 692 | 1.01 (0.98–1.04) | 1.04 (0.89–1.21) |  |
| Cardiac rehabilitation at discharge | |  |  |  |  | 0.75 |
|  | Yes | 104 | 1,049 | 1.00 (0.97–1.03) | 1.01 (0.87–1.16) |  |
|  | No | 84 | 400 | 0.99 (0.96–1.03) | 0.97 (0.80–1.17) |  |

BMI: body mass index, ACS: acute coronary syndrome, STEMI: ST-elevation myocardial infarction, NSTEMI: non-ST-elevation myocardial infarction, UA: unstable angina. Current smoking status had 1 missing value and cardiac rehabilitation status had 24 missing values

**Table S12.** Subgroup analysis of unadjusted risk of all-cause death per 1% and 5% weight loss

| Subgroup | | Events | n | HR per 1% (95% CI) | HR per 5% (95% CI) | Interaction p |
| --- | --- | --- | --- | --- | --- | --- |
| Sex | |  |  |  |  | 0.69 |
|  | Male | 100 | 1,250 | 1.02 (0.99–1.05) | 1.11 (0.95–1.30) |  |
|  | Female | 29 | 223 | 1.01 (0.96–1.06) | 1.05 (0.81–1.34) |  |
| Age group | |  |  |  |  | 0.38 |
|  | < 65 years | 36 | 901 | 1.02 (0.97–1.07) | 1.10 (0.88–1.39) |  |
|  | ≥ 65 years | 93 | 572 | 0.99 (0.95–1.03) | 0.97 (0.79–1.18) |  |
| BMI group | |  |  |  |  | 0.59 |
|  | < 27 kg/m^2^ | 48 | 492 | 1.03 (0.99–1.08) | 1.16 (0.93–1.44) |  |
|  | ≥ 27 kg/m^2^ | 81 | 981 | 1.01 (0.98–1.05) | 1.07 (0.90–1.27) |  |
| Current smoking | |  |  |  |  | 0.07 |
|  | Yes | 35 | 542 | 1.05 (1.00–1.10) | 1.28 (1.00–1.64) |  |
|  | No | 94 | 930 | 0.99 (0.96–1.03) | 0.97 (0.81–1.15) |  |
| ACS type |  |  |  |  |  | 0.67 |
|  | STEMI | 49 | 781 | 1.02 (0.97–1.06) | 1.08 (0.88–1.33) |  |
|  | NSTEMI / UA | 80 | 692 | 1.03 (0.99–1.06) | 1.14 (0.95–1.37) |  |
| Cardiac rehabilitation at discharge | |  |  |  |  | 0.56 |
|  | Yes | 58 | 1,049 | 1.03 (0.99–1.07) | 1.14 (0.94–1.38) |  |
|  | No | 67 | 400 | 1.05 (1.00–1.09) | 1.25 (1.00–1.55) |  |

BMI: body mass index, ACS: acute coronary syndrome, STEMI: ST-elevation myocardial infarction, NSTEMI: non-ST-elevation myocardial infarction, UA: unstable angina.

1 missing for current smoking, 24 missing for cardiac rehabilitation at discharge

Table S13. Achievement of secondary prevention cardiometabolic target per 1% and 5% weight loss

| N | Outcome and Cutoff | Model | OR (95% CI) | p-value |
| --- | --- | --- | --- | --- |
| 1,198 | LDL cholesterol <1.8 mmol/L | Unadjusted (1% WL) | 1.04 (1.02–1.06) | <0.001 |
|  |  | Unadjusted (5% WL) | 1.21 (1.1–1.32) |  |
|  |  | Adjusted (covariates) (1% WL) | 1.04 (1.02–1.06) | <0.001 |
|  |  | Adjusted (covariates) (5% WL) | 1.22 (1.11–1.35) |  |
|  |  | Adjusted (covariates and baseline value) (1% WL) | 1.04 (1.02–1.06) | <0.001 |
|  |  | Adjusted (covariates and baseline value) (5% WL) | 1.23 (1.11–1.36) |  |
|  | LDL cholesterol <1.4 mmol/L | Unadjusted (1% WL) | 1.02 (1–1.05) | 0.05 |
|  |  | Unadjusted (5% WL) | 1.12 (1–1.26) |  |
|  |  | Adjusted (covariates) (1% WL) | 1.03 (1–1.05) | 0.05 |
|  |  | Adjusted (covariates) (5% WL) | 1.13 (1–1.28) |  |
|  |  | Adjusted (covariates and baseline value) (1% WL) | 1.03 (1–1.05) | 0.04 |
|  |  | Adjusted (covariates and baseline value) (5% WL) | 1.15 (1.01–1.3) |  |
| 1,341 | Systolic BP < 130.0 mmHg | Unadjusted (1% WL) | 1.01 (0.99–1.03) | 0.18 |
|  |  | Unadjusted (5% WL) | 1.06 (0.97–1.15) |  |
|  |  | Adjusted (covariates) (1% WL) | 1.04 (1.02–1.06) | <0.001 |
|  |  | Adjusted (covariates) (5% WL) | 1.21 (1.1–1.34) |  |
|  |  | Adjusted (covariates and baseline value) (1% WL) | 1.04 (1.02–1.06) | <0.001 |
|  |  | Adjusted (covariates and baseline value) (5% WL) | 1.21 (1.1–1.34) |  |
|  | Systolic BP < 140.0 mmHg | Unadjusted (1% WL) | 1.01 (1–1.03) | 0.13 |
|  |  | Unadjusted (5% WL) | 1.07 (0.98–1.18) |  |
|  |  | Adjusted (covariates) (1% WL) | 1.04 (1.02–1.07) | <0.001 |
|  |  | Adjusted (covariates) (5% WL) | 1.24 (1.11–1.38) |  |
|  |  | Adjusted (covariates and baseline value) (1% WL) | 1.04 (1.02–1.07) | <0.001 |
|  |  | Adjusted (covariates and baseline value) (5% WL) | 1.24 (1.11–1.39) |  |
| 1,201 | Fasting glucose < 7.0 mmol/L | Unadjusted (1% WL) | 1.03 (1.01–1.06) | 0.01 |
|  |  | Unadjusted (5% WL) | 1.17 (1.03–1.32) |  |
|  |  | Adjusted (covariates) (1% WL) | 1.05 (1.02–1.09) | <.01 |
|  |  | Adjusted (covariates) (5% WL) | 1.29 (1.1–1.51) |  |
|  |  | Adjusted (covariates and baseline value) (1% WL) | 1.06 (1.02–1.09) | <.01 |
|  |  | Adjusted (covariates and baseline value) (5% WL) | 1.32 (1.12–1.55) |  |

WL: weight loss, LDL: low-density lipoprotein, BP: blood pressure, BMI: body mass index; N: number of available participant data, OR: odds ratio, CI: confidence interval

ORs represent the association between weight loss and the likelihood of achieving the 1-year target. ORs >1 indicate improved odds of target achievement with increasing weight loss

Estimates are shown per 1% and 5% weight loss, unadjusted, adjusted for covariates (sex, age, BMI, smoking, total cholesterol, hypertension, diabetes and eGFR) and adjusted for baseline values and covariates.

115 participants were excluded due to missing values in adjustment covariates. Summary of missing: smoking: 1, cholesterol: 93, eGFR: 37. Some participants had multiple missing. LDL-cholesterol status had 182 missing values, systolic BP status had 132 missing values, glucose status had 275 missing values.

Figure S1. Flowchart of Study Participants


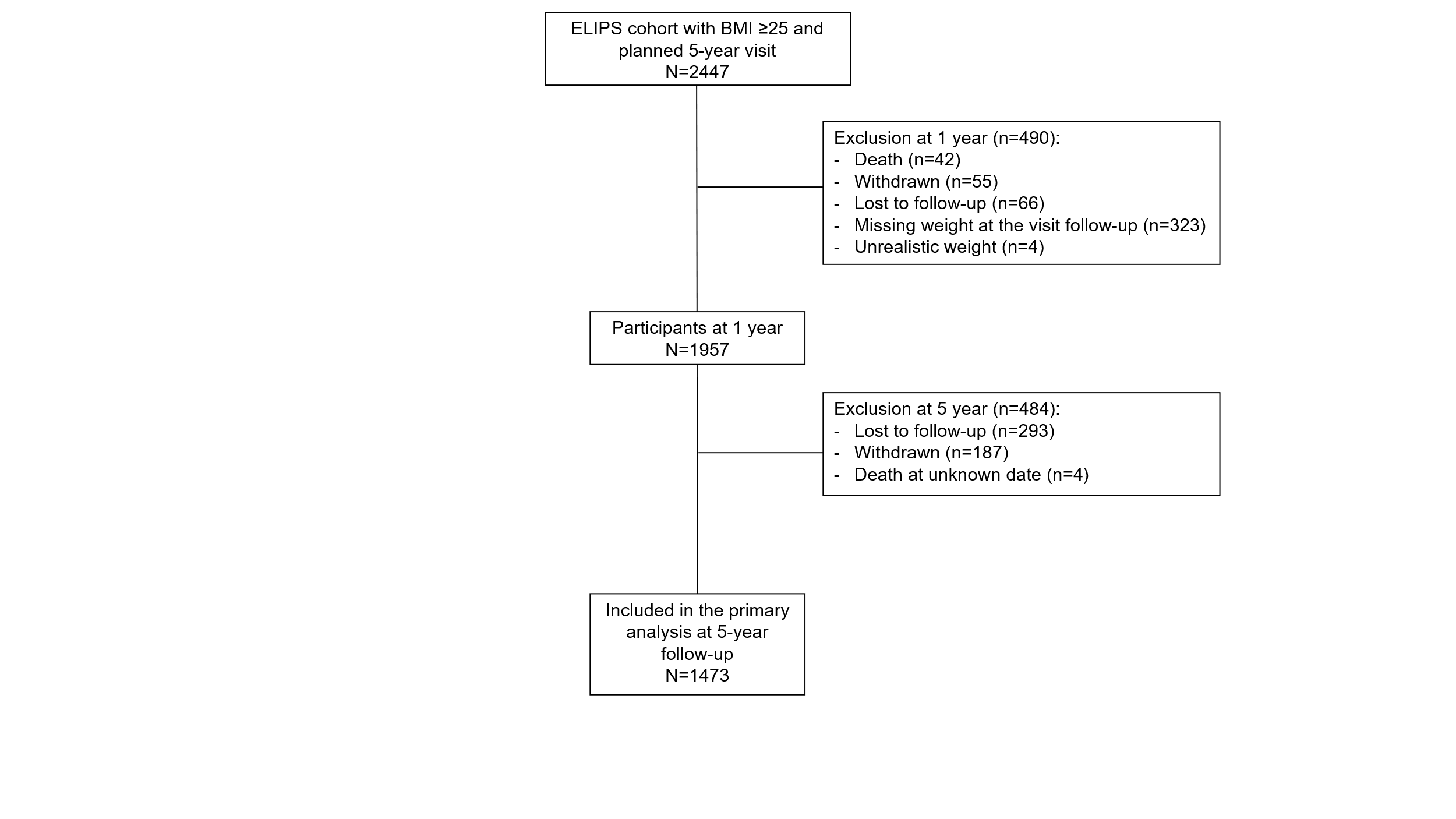


Figure S2. Weight Change Trajectories According to 1-year Weight Change Group


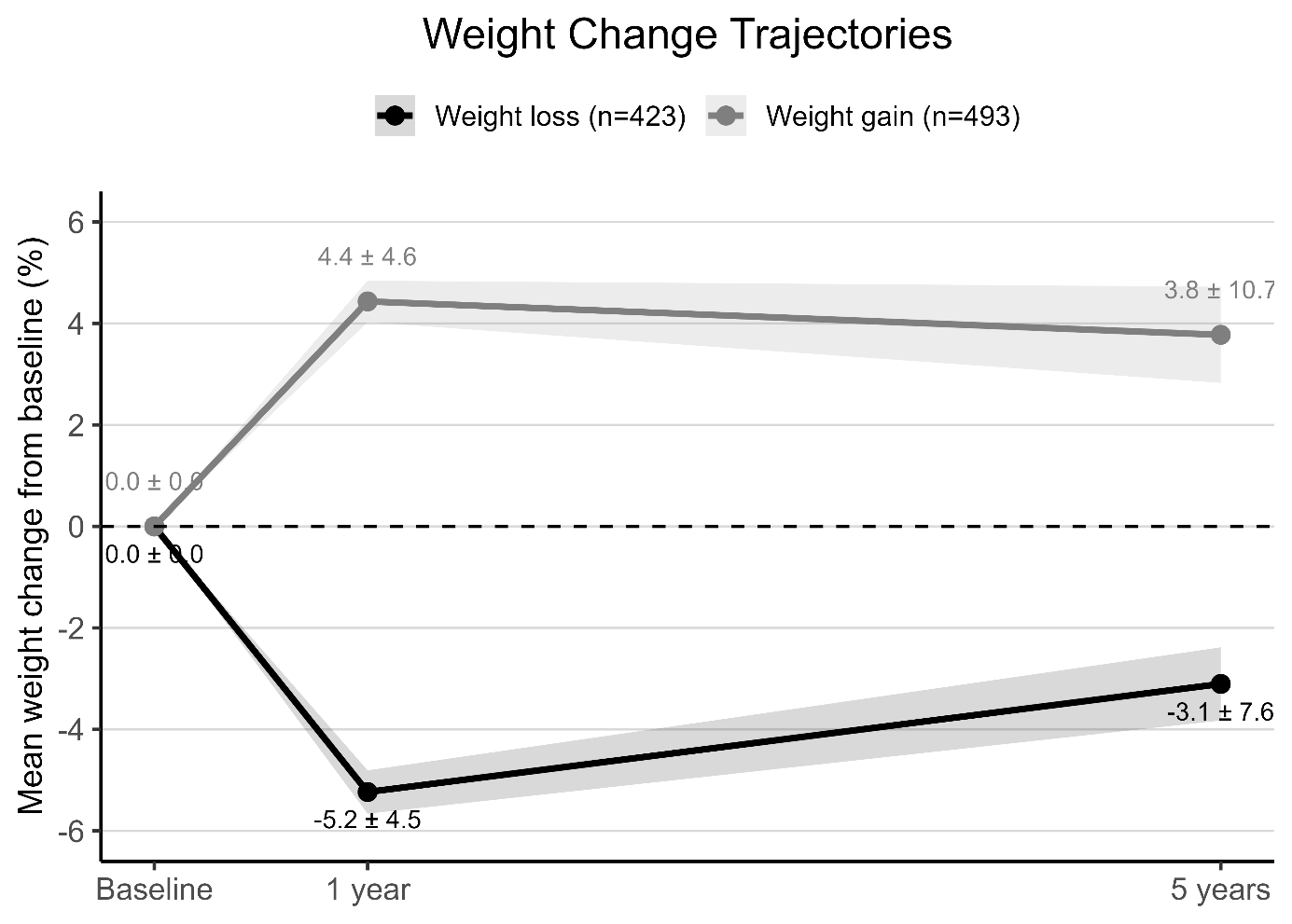


Lines represent mean percentage weight change from baseline, and shaded areas indicate 95% confidence intervals. The dashed horizontal line indicates no change from baseline. Only participants with available 5-year body weight measurements were included (n = 916)


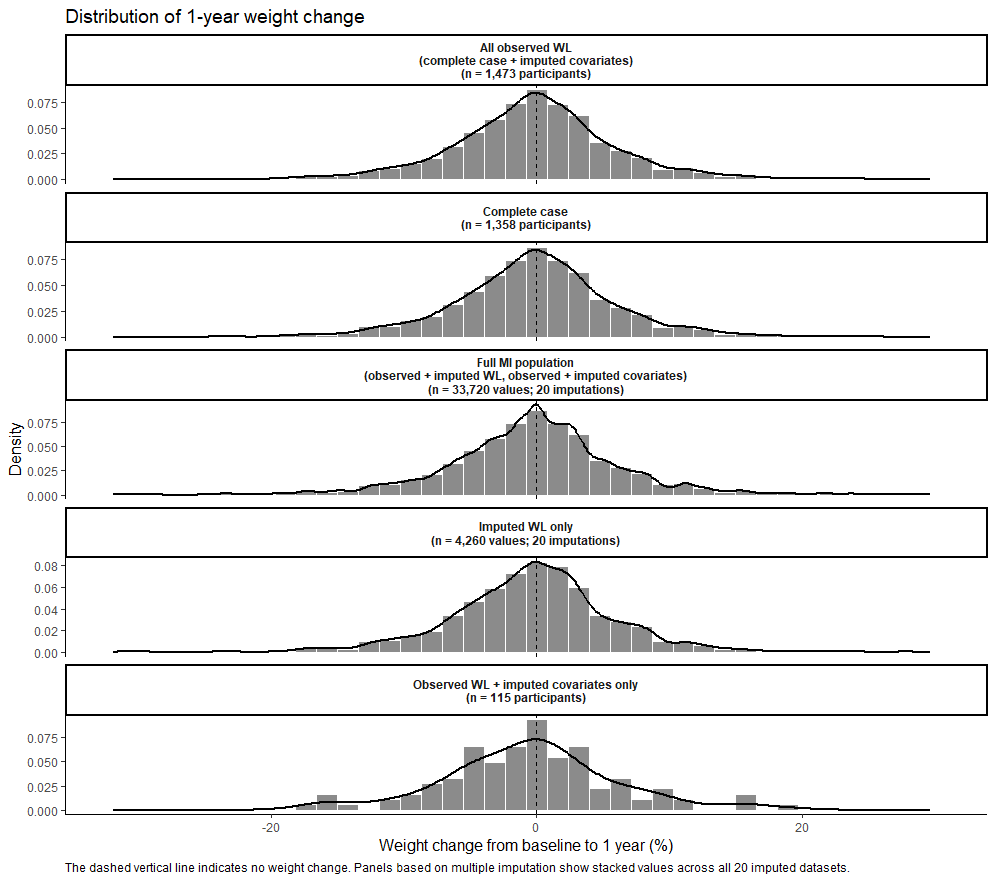
Figure S3. Distribution of 1-year weight change across complete-case and multiple imputation datasets

Histograms and density curves illustrate the distribution of percentage weight change from baseline to 1 year across complete-case participants, participants with observed weight change and imputed covariates, participants with imputed weight values, and the full multiple imputation dataset. The dashed vertical line indicates no weight change. Panels based on multiple imputation show stacked values across all 20 imputed datasets.

Figure S4. Adjusted Hazard Ratio for 3-point MACE by Percent Weight Change

**
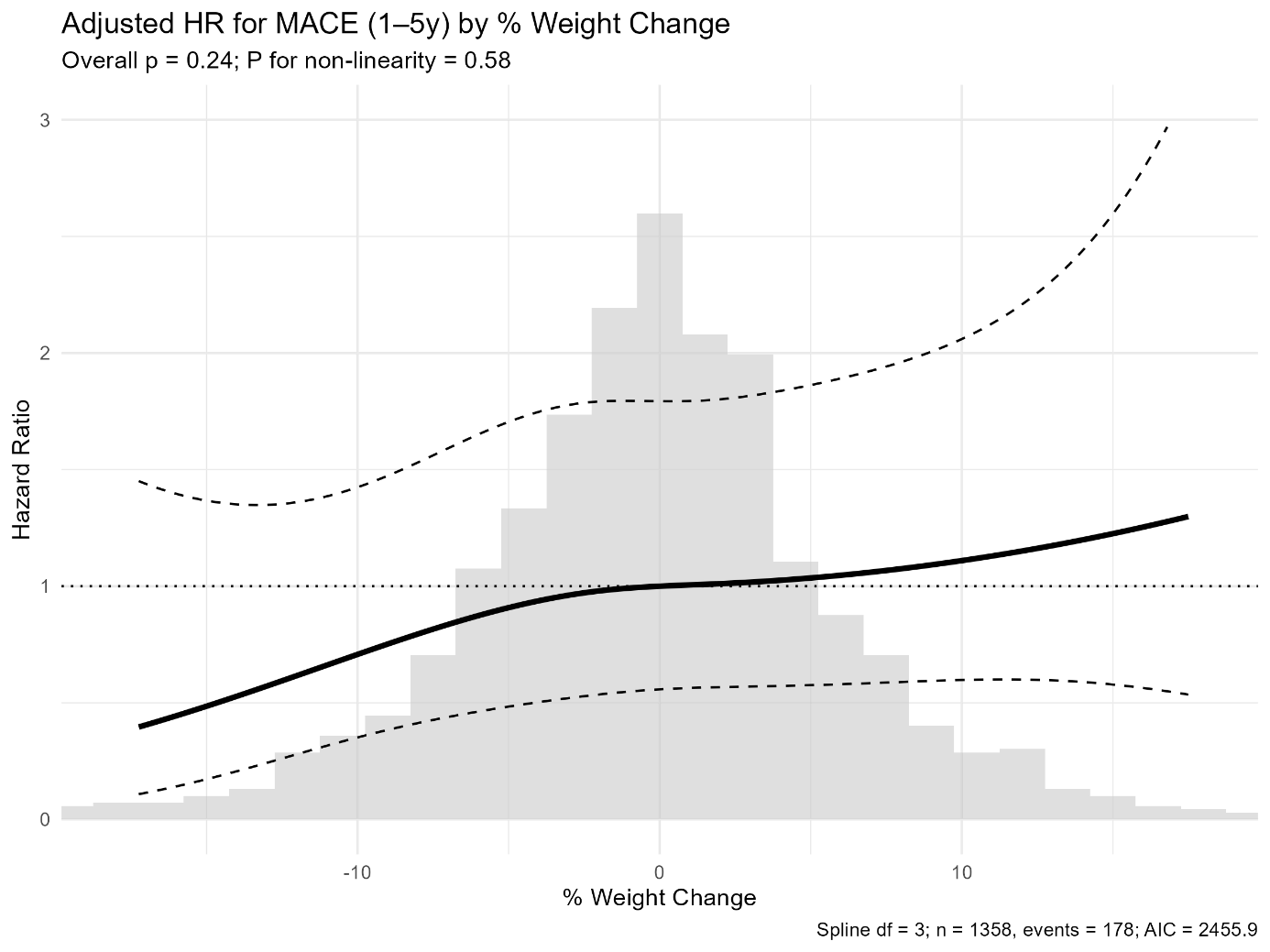
**

All models were adjusted for age, sex, baseline BMI, smoking status, total cholesterol, hypertension, diabetes, and eGFR. Overall p-value corresponds to the global significance of the spline term, and p for non-linearity reflects the comparison between spline and linear models. N = 1358, 115 participants were excluded due to missing values in adjustment covariates. Summary of missing: smoking: 1, cholesterol: 93, eGFR: 37. Some participants had multiple missing.

Figure S5. Adjusted Hazard Ratio for All-cause Death by Percent Weight Change


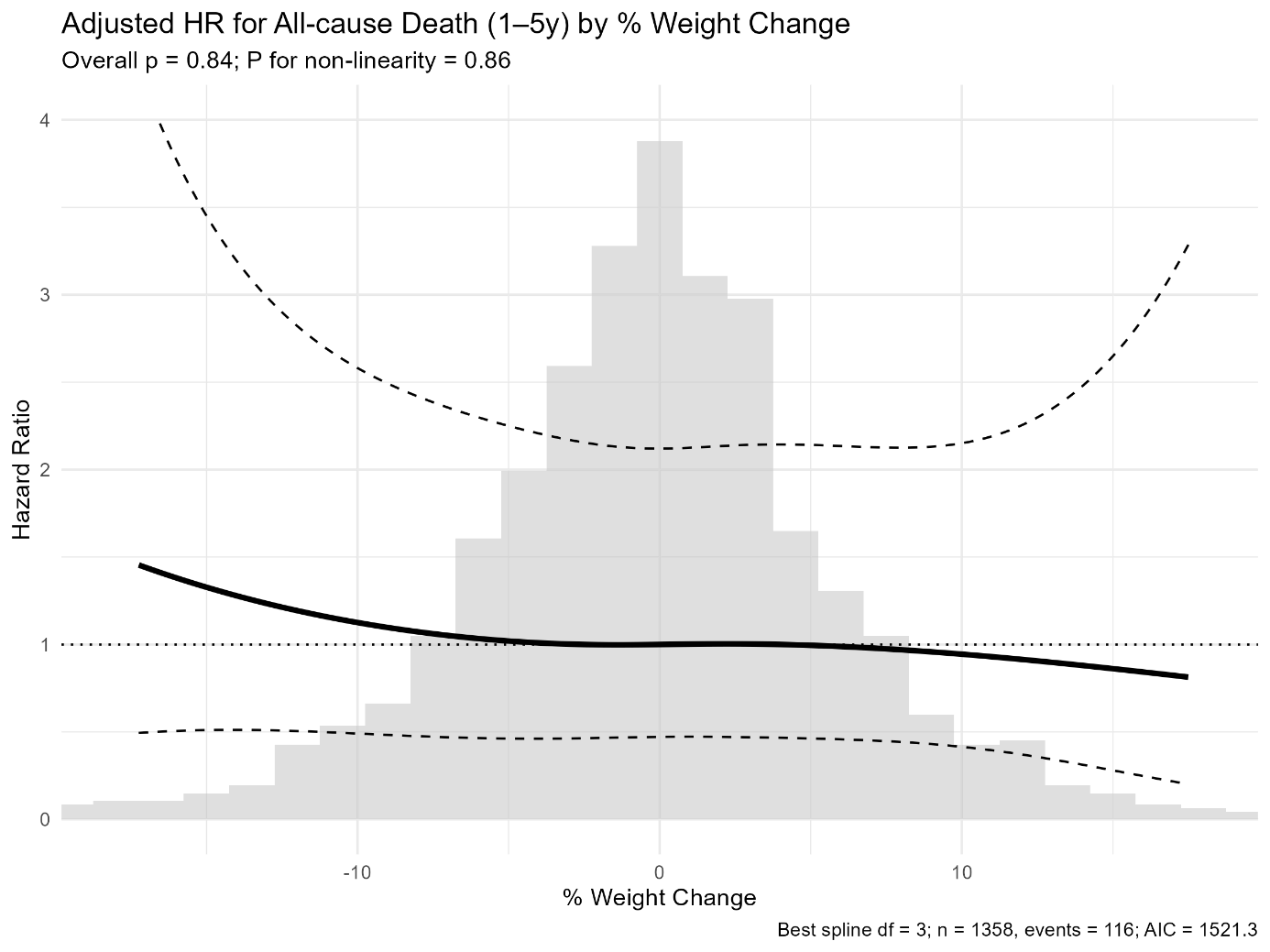


All models were adjusted for age, sex, baseline BMI, smoking status, total cholesterol, hypertension, diabetes, and eGFR. Overall p-value corresponds to the global significance of the spline term, and p for non-linearity reflects the comparison between spline and linear models. N = 1358, 115 participants were excluded due to missing values in adjustment covariates. Summary of missing: smoking: 1, cholesterol: 93, eGFR: 37. Some participants had multiple missing.

Figure S6. Adjusted Hazard Ratio for Cardiovascular Death by Percent Weight Change


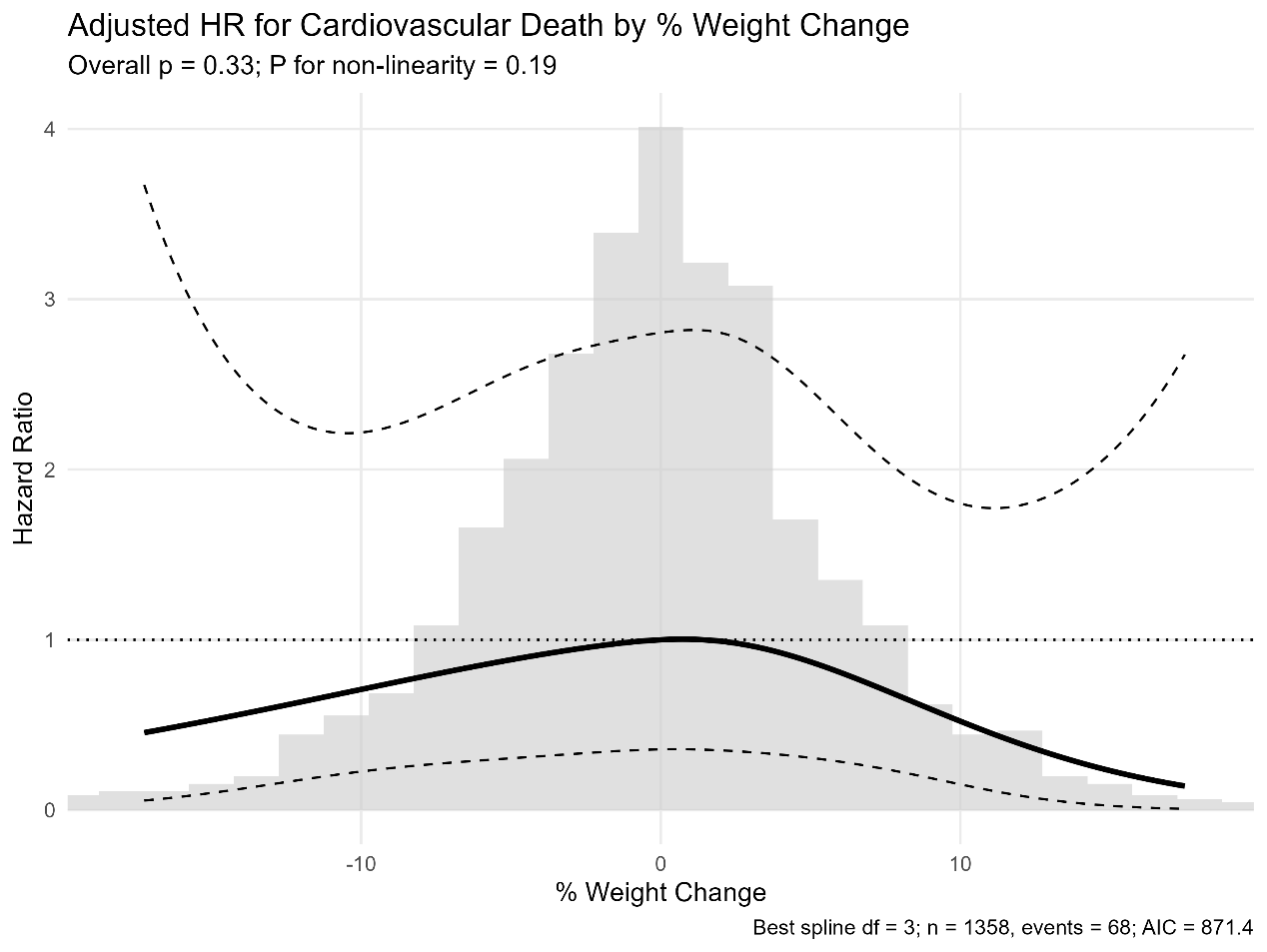


All models were adjusted for age, sex, baseline BMI, smoking status, total cholesterol, hypertension, diabetes, and eGFR. % weight change corresponds to the change between baseline and the 1-year visit. The event follow-up started 1-year after ACS. 115 participants were excluded due to missing values in adjustment covariates. Summary of missing: smoking: 1, cholesterol: 93, eGFR: 37. Some participants had multiple missing.

Figure S7. Adjusted Hazard Ratio for Myocardial Infarction by Percent Weight Change


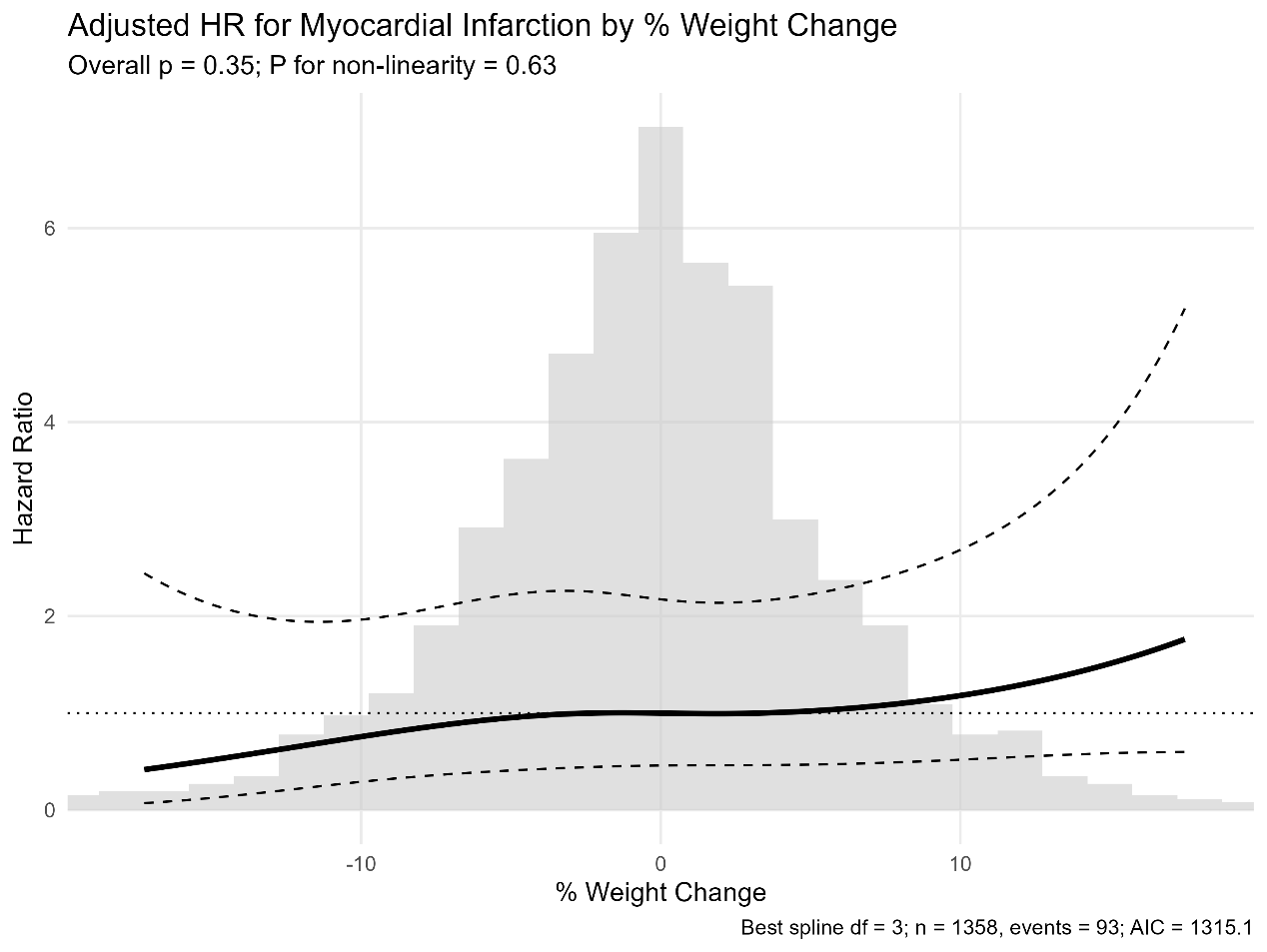


All models were adjusted for age, sex, baseline BMI, smoking status, total cholesterol, hypertension, diabetes, and eGFR. % weight change corresponds to the change between baseline and the 1-year visit. The event follow-up started 1-year after ACS. 115 participants were excluded due to missing values in adjustment covariates. Summary of missing: smoking: 1, cholesterol: 93, eGFR: 37. Some participants had multiple missing.

Figure S8. Adjusted Hazard Ratio for Coronary Revascularization by Percent Weight Change


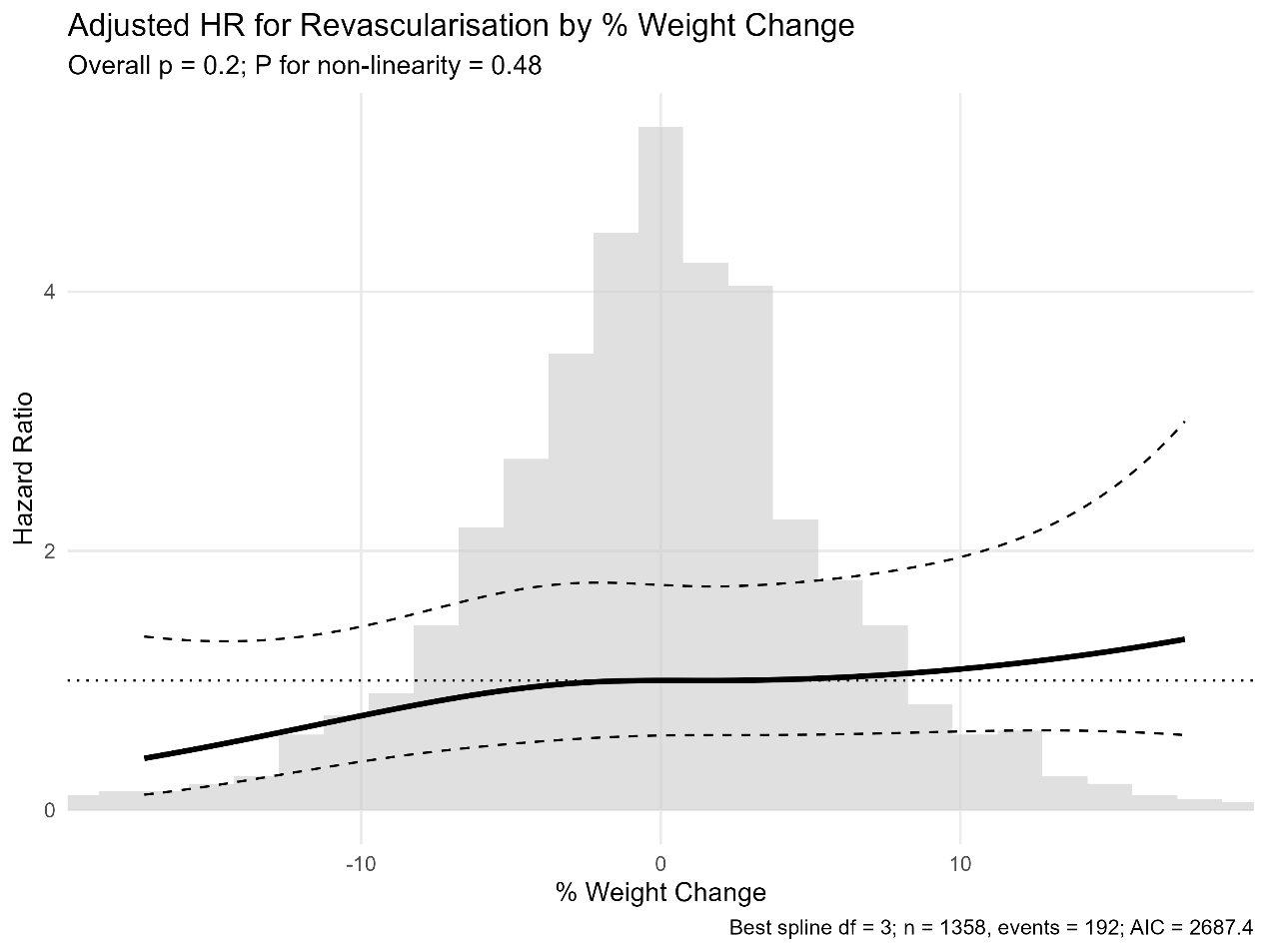


All models were adjusted for age, sex, baseline BMI, smoking status, total cholesterol, hypertension, diabetes, and eGFR. % weight change corresponds to the change between baseline and the 1-year visit. The event follow-up started 1-year after ACS. 115 participants were excluded due to missing values in adjustment covariates. Summary of missing: smoking: 1, cholesterol: 93, eGFR: 37. Some participants had multiple missing.

Figure S9. Adjusted Hazard Ratio for Stroke by Percent Weight Change


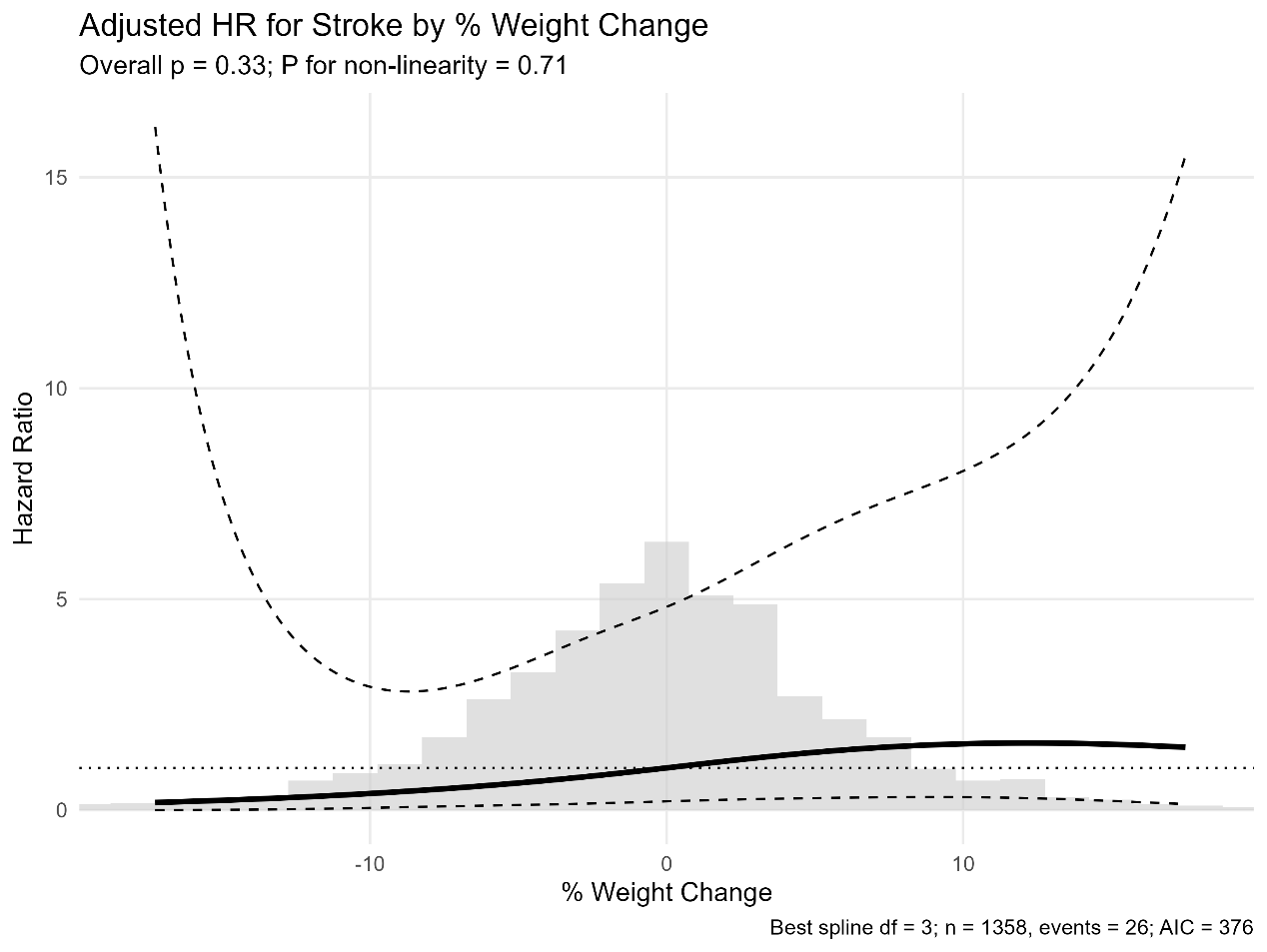


All models were adjusted for age, sex, baseline BMI, smoking status, total cholesterol, hypertension, diabetes, and eGFR. % weight change corresponds to the change between baseline and the 1-year visit. The event follow-up started 1-year after ACS. 115 participants were excluded due to missing values in adjustment covariates. Summary of missing: smoking: 1, cholesterol: 93, eGFR: 37. Some participants had multiple missing.

**Figure S10.** Adjusted Incidence Rate for 4-point MACE by Percent Weight Change

**
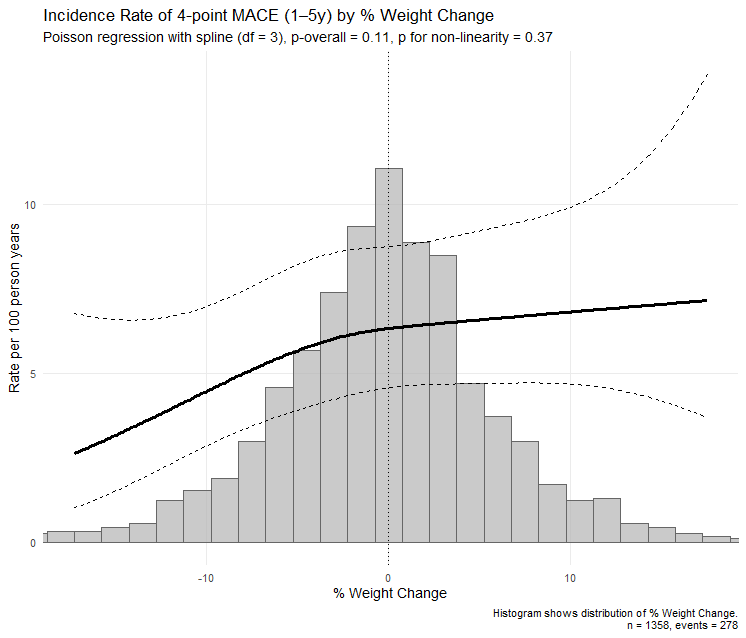
**

All models were adjusted for age, sex, baseline BMI, smoking status, total cholesterol, hypertension, diabetes, and eGFR. % weight change corresponds to the change between baseline and the 1-year visit. The event follow-up started 1-year after ACS. 115 participants were excluded due to missing values in adjustment covariates. Summary of missing: smoking: 1, cholesterol: 93, eGFR: 37. Some participants had multiple missing.

**Figure S11.** Adjusted Incidence Rate for 3-point MACE by Percent Weight Change

**
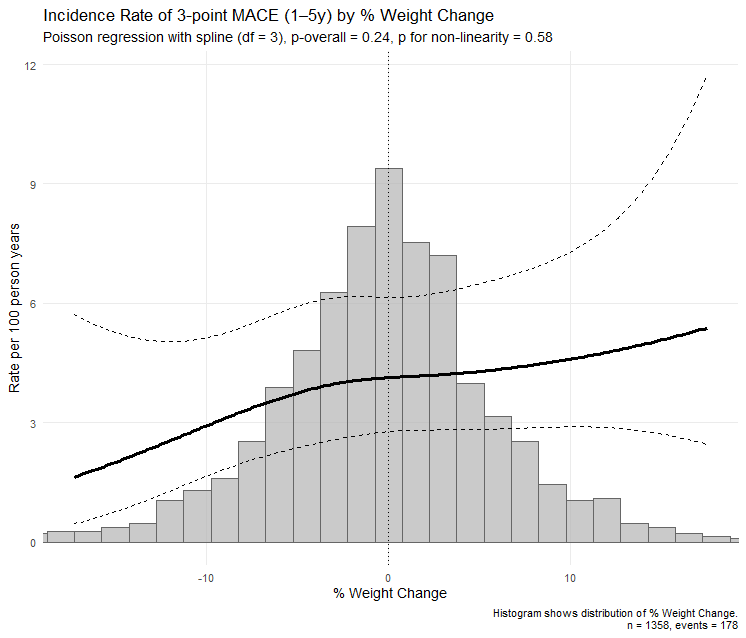
**

All models were adjusted for age, sex, baseline BMI, smoking status, total cholesterol, hypertension, diabetes, and eGFR. % weight change corresponds to the change between baseline and the 1-year visit. The event follow-up started 1-year after ACS. 115 participants were excluded due to missing values in adjustment covariates. Summary of missing: smoking: 1, cholesterol: 93, eGFR: 37. Some participants had multiple missing.

**Figure S12.** Adjusted Incidence Rate for All-cause Death by Percent Weight Change


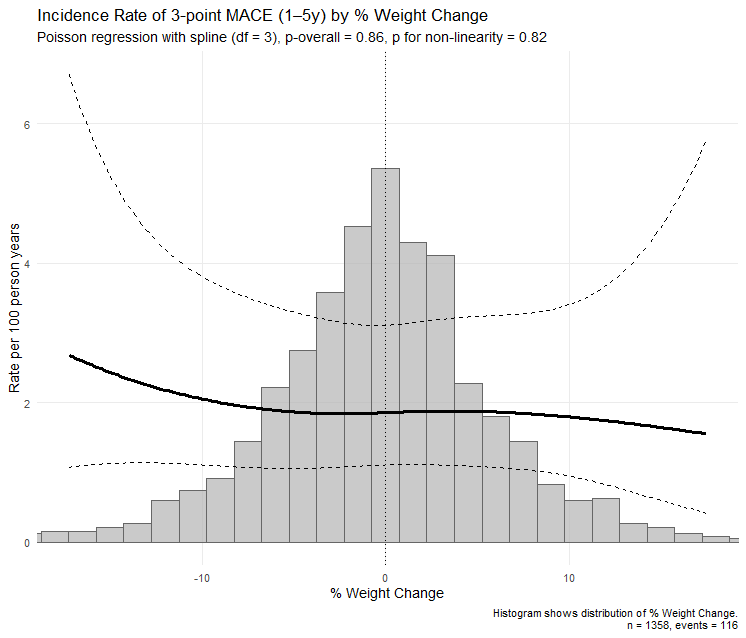


All models were adjusted for age, sex, baseline BMI, smoking status, total cholesterol, hypertension, diabetes, and eGFR. % weight change corresponds to the change between baseline and the 1-year visit. The event follow-up started 1-year after ACS. 115 participants were excluded due to missing values in adjustment covariates. Summary of missing: smoking: 1, cholesterol: 93, eGFR: 37. Some participants had multiple missing.
